# Pilot-scale purple phototrophic bacteria for wastewater treatment and resource recovery: an evidence-based framework for reactor selection and scale-up

**DOI:** 10.3389/fmicb.2026.1917392

**Published:** 2026-08-21

**Authors:** Sultan Shaikh, Gordon McKay

**Affiliations:** Division of Sustainable Development, College of Science and Engineering, Hamad Bin Khalifa University, Qatar Foundation, Doha, Qatar

**Keywords:** biohydrogen, bioplastics, circular economy, pilot-scale, purple non-sulfur bacteria, resource recovery, single-cell protein, wastewater treatment

## Abstract

Purple non-sulfur bacteria (PNSB), a major subgroup of purple phototrophic bacteria (PPB), have emerged as a promising platform for integrating wastewater treatment with resource recovery through the simultaneous production of value-added products, including single-cell protein (SCP), polyhydroxyalkanoates (PHAs), hydrogen, lipids, pigments, and other biomass-derived compounds. Although substantial progress has been achieved at laboratory scale, pilot-scale development remains fragmented across wastewater types, reactor configurations, operating conditions, and performance metrics, limiting cross-study comparison and practical technology selection. This review systematically synthesizes evidence from 15 pilot-scale studies ranging from 50 L to over 9,600 L, including flat-plate photobioreactors, tubular reactors, raceway ponds, membrane photobioreactors, integrated UASB-PPB systems, baffled reactors, and a stirred polypropylene-box photobioreactor operated under natural, filtered, or artificial illumination. Reported resource recovery outcomes include SCP contents of 42–65%, PHAs up to 36% of volatile suspended solids, hydrogen yields of 0.35–0.60 mol H₂ mol⁻¹ acetic acid, lipids up to 25%, and substantial pigment accumulation. Across these systems, *Rhodopseudomonas*, *Rhodobacter*, and *Blastochloris* consistently emerged as dominant genera supported by fermentative microbial consortia. The principal novelty of this review is the development of an evidence-based decision-support framework integrating wastewater characteristics, target products, reactor selection, technology readiness, and operational priorities. The framework identifies flat-plate photobioreactors as the preferred configuration for SCP production, integrated UASB–PPB systems for PHA recovery, and tubular and baffled photobioreactors for hydrogen production, while multi-criteria assessment ranks flat-plate photobioreactors, integrated UASB–PPB systems, and raceway ponds as the most promising configurations for pilot-scale implementation. The analysis further identifies light management, biomass harvesting, nutrient control, and process integration as the principal barriers to commercialization, providing practical guidance for reactor selection and accelerating the transition of PPB-based wastewater biorefineries toward commercial deployment.

## Graphical abstract

**Figure:**
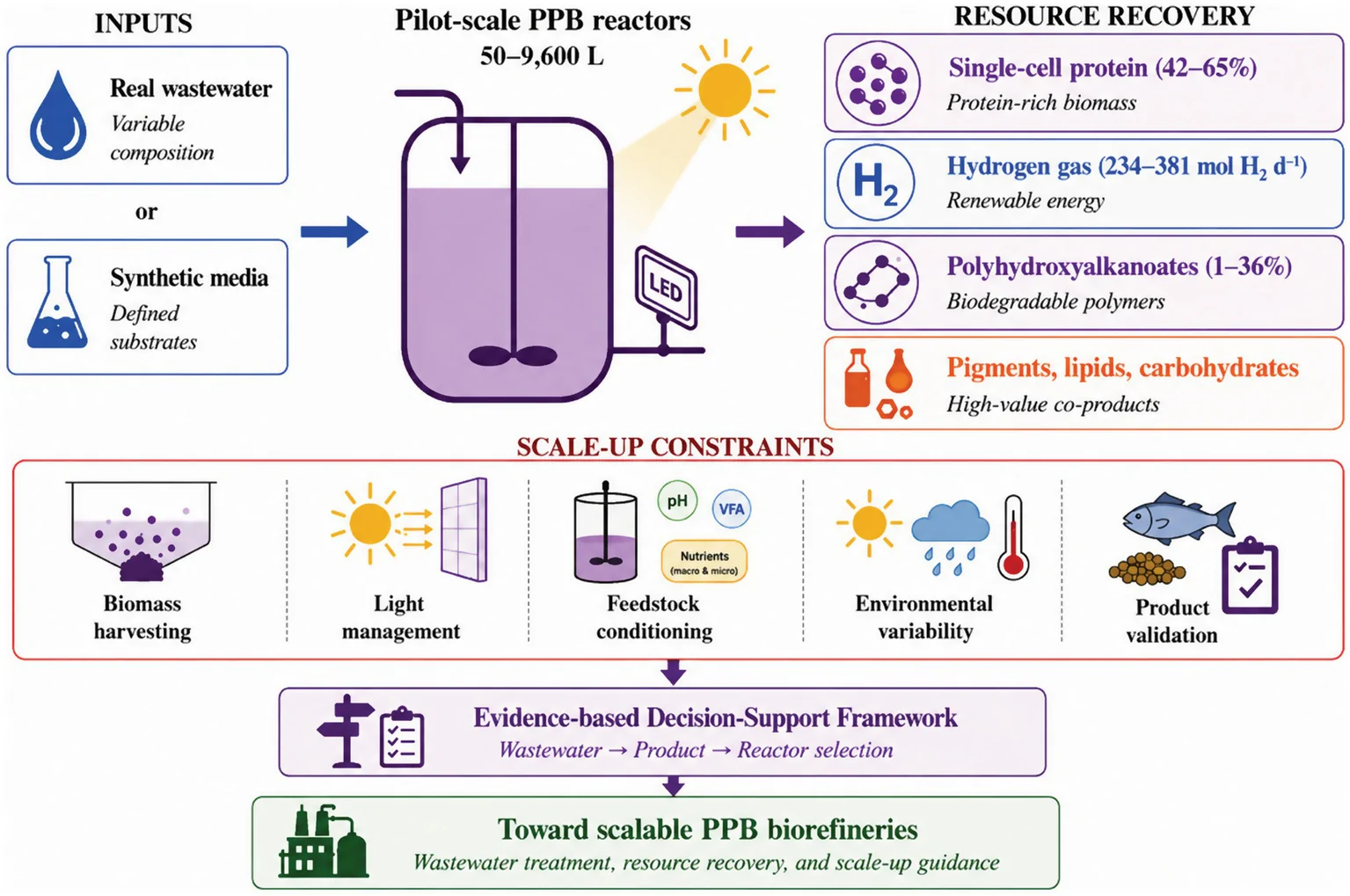

## Highlights

- Pilot-scale PPB recovered 42–65% SCP, 36% PHA, hydrogen, and pigments.
- Light distribution and biomass harvesting limit pilot-scale scale-up.
- Reactor engineering governs performance more than reactor size alone.
- Wastewater composition determines reactor selection and product recovery.
- FPPBRs and UASB-PPB systems show the highest scale-up potential.
- An evidence-based decision framework guides pilot-scale reactor selection and design.

## 1 Introduction

The increasing pressure on water resources, rising energy costs, and the need to decarbonize wastewater treatment have shifted research priorities from pollutant removal toward integrated treatment and resource recovery. Conventional wastewater treatment systems are energy-intensive and typically dissipate organic carbon and nutrients as waste sludge, rather than recovering them as value- added products (Molinos-Senante et al., 2024). In this context, biological technologies that enable simultaneous wastewater treatment and valorisation are receiving increasing attention within circular economy and bioresource recovery frameworks (Puyol et al., 2017b).

Purple non-sulfur bacteria (PNSB), a major subgroup of purple phototrophic bacteria (PPB), have emerged as a promising microbial platform for such applications due to their metabolic versatility and ability to grow photoheterotrophically under anaerobic or microaerobic conditions (Capson-Tojo et al., 2020; Zhang T. et al., 2024; Bayon-Vicente et al., 2025). PNSB can convert a wide range of organic substrates, particularly volatile fatty acids, into biomass and multiple value-added products, including single-cell protein (SCP), polyhydroxyalkanoates (PHAs), hydrogen gas, lipids, polysaccharides, pigments, coenzyme Q10, and 5-aminolevulinic acid (5-ALA) (Kim et al., 2012; Nunkaew et al., 2018; George et al., 2022; Shaikh et al., 2023a, 2023c, 2024b, 2025; Morrison and Bose, 2025; Ramadoss et al., 2026). Their capacity to integrate organic carbon removal with nutrient assimilation and product formation has led to extensive laboratory-scale research and an increasing number of pilot-scale demonstrations across diverse reactor configurations and wastewater types (Carlozzi and Sacchi, 2001; Carlozzi et al., 2006; Boran et al., 2010, 2012a, 2012b; Gebicki et al., 2010; Adessi et al., 2012; Zhang et al., 2017; Lu et al., 2019a; Hülsen et al., 2022a, 2022b; Capson-Tojo et al., 2023b; Wada et al., 2023b; Almeida et al., 2024; Doki et al., 2024).

Recent reviews describe PPB as metabolically flexible platforms for integrated wastewater treatment and resource recovery, with strong emphasis on their operation in different photobioreactor configurations. These studies report that PPB can efficiently assimilate organic carbon and nutrients from real wastewaters under photoheterotrophic and mixed growth modes, while producing value-added products such as single-cell protein (SCP), hydrogen, polyhydroxyalkanoates (PHAs), pigments, lipids, polysaccharides, 5-ALA, and coenzyme Q10 (Lu et al., 2019b; Cao et al., 2020; Capson-Tojo et al., 2020; Sali and Mackey, 2021; Rashid et al., 2022; Wada et al., 2022). These reviews discuss a range of photobioreactor designs, including closed tubular, flat-plate, and open systems, and highlight the importance of light wavelength and intensity, oxygen control, and hydraulic retention time for stable operation and scalability (Adessi and De Philippis, 2014; Shaikh et al., 2023b). In addition, broader applications in sustainable agriculture and environmental remediation are addressed, such as plant growth promotion, greenhouse gas mitigation, and detoxification of heavy metals and hazardous organic pollutants (Sundar and Chao, 2022; Dhar et al., 2023). While these studies provide valuable insights, they primarily focus on laboratory-scale systems or single-product optimization and therefore do not fully address the engineering, operational, and systems-level challenges associated with pilot-scale operation and scale-up. Pilot-scale studies are a critical step toward full-scale implementation because they enable the identification of scale-up constraints, validation of reactor performance under realistic operating conditions, optimization of process integration and operational strategies, and assessment of long-term process stability prior to commercial deployment.

A pilot-scale perspective is adopted in this review, focusing exclusively on systems with working volumes of 50 L and above, where many of the engineering and operational constraints governing industrial implementation first become evident. It provides the first comprehensive synthesis of pilot-scale PPB systems across diverse wastewater types and comparatively evaluates reactor performance according to wastewater characteristics, pretreatment requirements, illumination strategies, microbial community dynamics, treatment performance, and resource recovery outcomes. In addition to critically evaluating the available literature, this review develops a comparative framework for reactor selection based on wastewater characteristics and target products, supported by technology readiness assessment, and commercialization considerations. The objective is to provide practical guidance for researchers and engineers seeking to select, design, and scale PPB-based systems for wastewater treatment and resource recovery.

## 2 Review methodology

### 2.1 Literature search and study selection

Published pilot-scale studies on the application of Purple Phototrophic Bacteria (PPB) for wastewater treatment and resource recovery conducted between 2000 and 2025 were systematically identified through searches of Scopus, Web of Science, and Google Scholar. Search terms included combinations of “purple non-sulfur bacteria,” “pilot-scale,” “photobioreactor,” “wastewater treatment,” “resource recovery,” “outdoor cultivation,” “photosynthetic bacteria,” and “*Rhodopseudomonas*.” Additional relevant studies were identified through backward citation tracking of the retrieved publications.

Studies were included if they met the following criteria: (i) reactor working volume ≥50 L; (ii) operation under natural sunlight, artificial illumination, or a combination of both; (iii) use of PPB or PPB-enriched mixed cultures as the dominant microbial community; and (iv) application to real, fermented, or synthetic wastewaters or cultivation media relevant to process scale-up and resource recovery. Laboratory-scale studies (<50 L), non-phototrophic biological systems, conference abstracts, and review articles were excluded.

For each eligible study, data were systematically extracted on reactor configuration, reactor geometry, operating conditions, illumination strategy, wastewater or cultivation medium, pretreatment requirements, treatment performance, biomass and product recovery, and key operational challenges. Where appropriate, reported values were normalized to facilitate consistent comparison of reactor scale, treatment efficiency, resource recovery performance, and operational characteristics across studies.

### 2.2 Development of the evidence-based decision-support framework

The extracted evidence was synthesized to develop an evidence-based decision-support framework for pilot-scale PPB reactor selection and scale-up. Reactor configurations were systematically evaluated using standardized criteria representing treatment performance, resource recovery potential, scale-up readiness, energy efficiency, operational simplicity, harvesting characteristics, and economic considerations. These criteria were selected to capture the principal technical and operational factors influencing reactor selection for different wastewater types and target products.

The synthesized evidence informed the development of the weighted multi-criteria ranking, technology readiness level (TRL) assessment, and qualitative economic comparison. The framework was designed to support reactor selection according to wastewater characteristics, target products, and operational priorities rather than to provide absolute quantitative predictions. Detailed scoring criteria, weighting methodology, TRL assignment, and the evidence supporting the qualitative economic comparison are provided in Supplementary methods S7–S9.

## 3 Overview of pilot-scale systems

Pilot-scale investigations play a critical role in bridging laboratory findings and real-world implementation by evaluating operational stability, scalability, and resource recovery potential under realistic conditions. Within wastewater treatment and valorisation research, PNSB have emerged as a promising functional group due to their metabolic flexibility, tolerance to variable wastewater characteristics, and the ability to simultaneously remove pollutants, while generating value-added bioproducts (Capson-Tojo et al., 2020; Bayon-Vicente et al., 2025). This section provides an overview of the pilot-scale systems reported to date, highlighting key differences in reactor configuration, operational scale, illumination strategy, wastewater characteristics, and resource recovery objectives. A consolidated summary of the reviewed pilot-scale studies is provided in Table 1.

**Table 1:** Overview of pilot-scale photobioreactor studies for wastewater treatment and resource recovery.

Study	Country	Reactor type	Reactor volume	Operating period (days)	Illumination	Wastewater/medium	Resources recovered	Culture type
Almeida et al. (2024)	Spain	UASB + PPB ponds	2 × 9,600 L	540	Natural sunlight	Fermented domestic wastewater with molasses	PHA	PPB-enriched mixed culture
Doki et al. (2024)	India	Raceway photobioreactor	2,500 L	5	Artificial light	Fermented sewage	SCP and lipids	PPB-enriched mixed culture
Wada et al. (2023b)	Qatar	Stirred polypropylene-box photobioreactor	65 L	36	Artificial light	Fuel synthesis wastewater	SCP, lipids, Crts, and BChls	PPB-enriched mixed culture
Capson-Tojo et al. (2023b)	Australia	Flat-plate photobioreactor	953 L	78	Natural sunlight (UV–VIS filtered)	Pre-fermented poultry-processing wastewater	SCP	PPB-enriched mixed culture
Hülsen et al. (2022b)	Australia	Flat-plate photobioreactor	950 L	253	Natural sunlight (NIR filtered)	Poultry-processing wastewater (raw and fermented DAF effluent)	SCP	PPB-enriched mixed culture
Hülsen et al. (2022a)	Australia	Flat-plate photobioreactor	60 L, 80 L, and 100 L	90 (Piggery WW) and 214 (Chicken WW)	Natural sunlight (IR filtered and full spectrum)	Pre-settled piggery wastewater and meat chicken processing wastewater	SCP, Crts, and BChls	PPB-enriched mixed culture
Lu et al. (2019a)	China	Membrane photobioreactor	240 L	440	Artificial and natural sunlight	Brewery wastewater	SCP, polysaccharides, Crts, BChls, and coenzyme Q10	PPB-enriched mixed culture
Zhang et al. (2017)	China	Baffled photobioreactor	4,000 L	Not reported	Natural sunlight	Synthetic glucose based medium	H₂	PPB-enriched mixed culture
Adessi et al. (2012)	Italy	Horizontal tubular photobioreactor	50 L	21	Natural sunlight	RPP synthetic medium	H₂	Pure culture (*Rhodopseudomonas palustris strain 42OL*)
Boran et al. (2010)	Turkey	Tubular photobioreactor	80 L	30	Artificial light	Modified BP medium (acetate, glutamate, and phosphate buffer)	H₂	Pure culture (*Rhodobacter capsulatus* DSM 1710)
Boran et al. (2012a)	Turkey	Tubular photobioreactor	90 L	16	Artificial and natural sunlight	Modified BP medium (acetate, glutamate, and phosphate buffer)	H₂	Pure culture (*Rhodobacter capsulatus* YO3, Hup^-^)
Boran et al. (2012b)	Turkey	Tubular photobioreactor	90 L	20	Artificial and natural sunlight	Thick juice dark fermenter effluent	H₂	Pure culture (*Rhodobacter capsulatus* DSM 1710)
Gebicki et al. (2010)	Germany	Tubular and Flat-plate photobioreactor	65 L and 100 L, respectively	25	Natural sunlight	Acetic acid, sodium lactate, and sodium glutamate	H₂	Pure culture (*Rhodobacter* *capsulatus* DSM 155)
Carlozzi et al. (2006) and Carlozzi and Sacchi (2001)	Italy	Underwater tubular photobioreactor (UwTP)	53 L	84 and 180, respectively	Natural sunlight	Modified van Niel synthetic medium (sodium acetate, ammonium chloride, and yeast extract)	PHB, SCP, Crts, and BChls	Pure culture (*Rhodopseudomonas palustris strain 42OL*)

PPB, purple phototrophic bacteria; UASB, upflow anaerobic sludge blanket; SCP, single-cell protein; PHA, polyhydroxyalkanoates; PHB, polyhydroxybutyrate; Crts, carotenoids; BChls, bacteriochlorophylls; DAF, dissolved air flotation; WW, wastewater; UwTP, underwater tubular photobioreactor; UV, ultraviolet; VIS, visible; NIR, near-infrared; IR, infrared; RPP, *Rhodopseudomonas palustris* producing growth medium; BP, Biebl and Pfennig growth medium.

The reported systems span a broad range of scales, from approximately 50 L to more than 9,600 L, reflecting different approaches to process intensification and scale-up. Large outdoor installations, such as integrated UASB-phototrophic pond systems, have demonstrated the feasibility of continuous wastewater treatment and resource recovery under realistic operating conditions. In contrast, smaller pilot systems have primarily focused on process optimisation, microbial enrichment, and targeted production of high-value products such as hydrogen, polyhydroxyalkanoates, and microbial protein (Adessi et al., 2012; Almeida et al., 2024; Boran et al., 2012a, 2012b; Boran et al., 2010; Gebicki et al., 2010).

Illumination remains one of the most important operational variables in pilot-scale PPB systems. Outdoor reactors predominantly rely on natural sunlight, often supplemented with optical filtering approaches to enhance selective PPB enrichment, whereas indoor systems employ artificial light sources to maintain controlled irradiance and spectral quality (Capson-Tojo et al., 2023a; Doki et al., 2024; Hülsen et al., 2022a, 2022b; Wada et al., 2023a). These differences in light management have important implications for treatment performance, product recovery, energy demand, and scalability.

A wide variety of wastewater streams have been investigated, including domestic wastewater, fermented sewage, poultry-processing wastewater, piggery wastewater, brewery wastewater, fuel synthesis wastewater, and dark-fermenter effluents. Several studies also employed synthetic media to evaluate specific metabolic pathways under controlled conditions (Almeida et al., 2024; Boran et al., 2012b; Boran et al., 2012a; Boran et al., 2010; Carlozzi et al., 2006; Carlozzi and Sacchi, 2001; Doki et al., 2024; Hülsen et al., 2022b; Hülsen et al., 2022a; Lu et al., 2019a; Wada et al., 2023b; Zhang et al., 2017). The diversity of wastewater characteristics, reactor configurations, and operational strategies highlights that no single pilot-scale technology is universally optimal. Instead, system performance depends strongly on the interaction between wastewater composition, process objectives, and reactor design. This observation provides the basis for the comparative assessment and reactor selection framework developed in later sections of this review.

## 4 Wastewater types and pretreatment strategies

The type and composition of wastewater play a critical role in determining the growth performance and metabolic response of PPB during pilot-scale applications. Owing to their metabolic versatility, PPB can utilize a wide spectrum of organic substrates under anaerobic, microaerobic, or even aerobic-dark conditions (Amini et al., 2025a; Cerruti et al., 2023). However, successful cultivation and resource recovery depend largely on biodegradable organic carbon availability, nutrient balance (C/N/P), and the absence of inhibitory compounds. Wastewaters rich in readily biodegradable organics generally favor photoheterotrophic growth and biomass production, whereas nutrient-deficient, saline, or toxic wastewaters often require pretreatment or nutrient supplementation to achieve stable reactor performance (Sali and Mackey, 2021). Supplementary Table S1 summarizes the physicochemical characteristics of the wastewater streams investigated in the reviewed pilot-scale studies, including COD, nitrogen, phosphorus, pH, and indicative C/N and C/P ratios where sufficient data were available. The comparison demonstrates substantial variability in wastewater composition, highlighting that nutrient balance rather than organic loading alone governs PPB enrichment and resource recovery potential. Besides overall nutrient ratios, substrate composition strongly influences PPB metabolism. VFA-rich wastewaters generally favor biomass accumulation and bioproduct synthesis, whereas complex organic substrates often require pretreatment to improve biodegradability (Zhang et al., 2014; Cardeña et al., 2016). Likewise, nitrogen availability regulates metabolic pathways, with nitrogen-rich wastewaters promoting biomass and protein production, while nitrogen limitation favors intracellular storage compounds and hydrogen production (Li et al., 2010; Shaikh et al., 2024c). These differences emphasize that wastewater characteristics should be matched with the intended resource recovery objective when selecting pilot-scale PPB systems.

### 4.1 Domestic wastewater

Domestic wastewater and fermented sewage were investigated by Almeida et al. (2024) and Doki et al. (2024). As summarized in Supplementary Table S1, these wastewaters are characterized by relatively low organic carbon availability and moderate nutrient concentrations, which can limit PPB biomass productivity. Consequently, upstream fermentation or supplementary carbon addition may be beneficial to increase VFA availability and improve resource recovery performance.

### 4.2 High-strength organic and industrial wastewater

High-strength industrial wastewaters, including poultry-processing wastewater (Hülsen et al., 2022b; Capson-Tojo et al., 2023b), fuel synthesis wastewater (Wada et al., 2023b), and dark-fermenter effluents (Boran et al., 2012b), are representative examples of high-strength organic waste streams, which are characterized by elevated organic loading and variable nutrient composition (Hamza et al., 2016) (Supplementary Table S1). Carbon-rich streams such as fuel synthesis wastewater and dark-fermenter effluents are promising substrates for hydrogen and PHA production because of their high biodegradable carbon content. However, their limited nitrogen and phosphorus availability often necessitates nutrient supplementation and pH control to sustain PPB growth. In contrast, poultry-processing wastewater provides a more balanced nutrient composition, making it suitable for simultaneous wastewater treatment, biomass production, and nutrient recovery.

### 4.3 Animal wastewater

Animal wastewaters, particularly piggery and poultry-processing wastewaters (Hülsen et al., 2022a), are among the most nutrient-rich substrates evaluated in pilot-scale PPB systems (Supplementary Table S1). Their high nitrogen and phosphorus contents support microbial protein production and nutrient recovery but may also result in ammonia inhibition under high-strength conditions. Therefore, successful operation often requires dilution or process control strategies to balance nutrient availability while minimizing inhibitory effects.

### 4.4 Brewery wastewater

Brewery wastewater has a relatively stable composition with moderate organic loading and comparatively low suspended solids (Supplementary Table S1), making it one of the more straightforward wastewaters for pilot-scale PPB cultivation (Lu et al., 2019a). Its balanced characteristics reduce pretreatment requirements and support stable biomass production, highlighting its potential as a reliable feedstock for microbial protein production.

### 4.5 Synthetic media

Synthetic media were primarily employed in pure-culture studies to investigate PPB physiology and optimize hydrogen or PHA production under controlled conditions (Carlozzi et al., 2006; Boran et al., 2010, 2012a; Gebicki et al., 2010; Adessi et al., 2012; Zhang et al., 2017). Although these systems are valuable for understanding metabolic behavior, their reliance on refined substrates limits their practical relevance for pilot-scale wastewater treatment. Consequently, results obtained using synthetic media should be interpreted as indicators of biological potential rather than predictors of full-scale process performance.

### 4.6 Implications for reactor selection

The reviewed wastewater streams demonstrate that feedstock characteristics directly influence reactor selection and resource recovery objectives. Carbon-rich but nutrient-deficient wastewaters are generally better suited to closed photobioreactor systems, where nutrient supplementation and environmental conditions can be carefully controlled. Conversely, nutrient-rich wastewaters, such as poultry-processing and piggery wastewaters, favor biomass-oriented systems that exploit their inherent nitrogen and phosphorus content for microbial protein production and nutrient recovery. Domestic and brewery wastewaters occupy an intermediate position and can support multiple recovery pathways with relatively low operational complexity. These observations highlight that wastewater composition should be evaluated alongside reactor design and desired products when selecting pilot-scale PPB technologies.

### 4.7 Pretreatment strategies

In pilot-scale PPB systems, influent pretreatment is a critical upstream step that improves substrate availability, light penetration, nutrient balance, and reactor stability, thereby enhancing microbial activity and resource recovery. The choice of pretreatment depends on wastewater characteristics and the intended recovery objective. Supplementary Table S2 summarizes the pretreatment strategies reported in the reviewed pilot-scale studies, including wastewater types, unit operations, their functions, and key performance indicators.

Pretreatment strategies varied according to wastewater composition. Domestic wastewater primarily underwent solids and grease removal through screening or filtration, whereas fermented municipal sewage additionally employed acidogenic fermentation, clarification, and pH adjustment to increase VFA availability before PPB cultivation (Almeida et al., 2024; Doki et al., 2024). Poultry-processing wastewater generally required dissolved air flotation (DAF) followed by acidogenic fermentation to remove suspended solids and convert complex organics into VFAs, thereby improving substrate quality for PPB growth (Hülsen et al., 2022b; Capson-Tojo et al., 2023b). In contrast, thick juice dark-fermenter effluent required only dilution and pH adjustment because it already contained soluble fermentation products (Boran et al., 2012b). Piggery wastewater was typically pretreated by settling to remove coarse solids, while acetate supplementation was occasionally applied to compensate for insufficient VFA concentrations (Hülsen et al., 2022a). Brewery wastewater required only simple gravity settling because of its relatively low suspended solids content (Lu et al., 2019a), whereas fuel synthesis wastewater required primarily pH adjustment owing to its acidic nature rather than solids removal (Shaikh et al., 2023c, 2024a; Wada et al., 2023a).

Overall, pretreatment primarily served three functions: increasing biodegradable carbon through fermentation, reducing suspended solids to improve light penetration, and optimizing pH and nutrient conditions for PPB enrichment. Consequently, pretreatment should be selected according to wastewater characteristics and the desired resource recovery pathway while balancing improvements in process performance against the additional capital and operating costs associated with fermentation, solids removal, nutrient supplementation, and pH adjustment.

## 5 Photobioreactor design, scale, and illumination strategies

**Figure 1:**
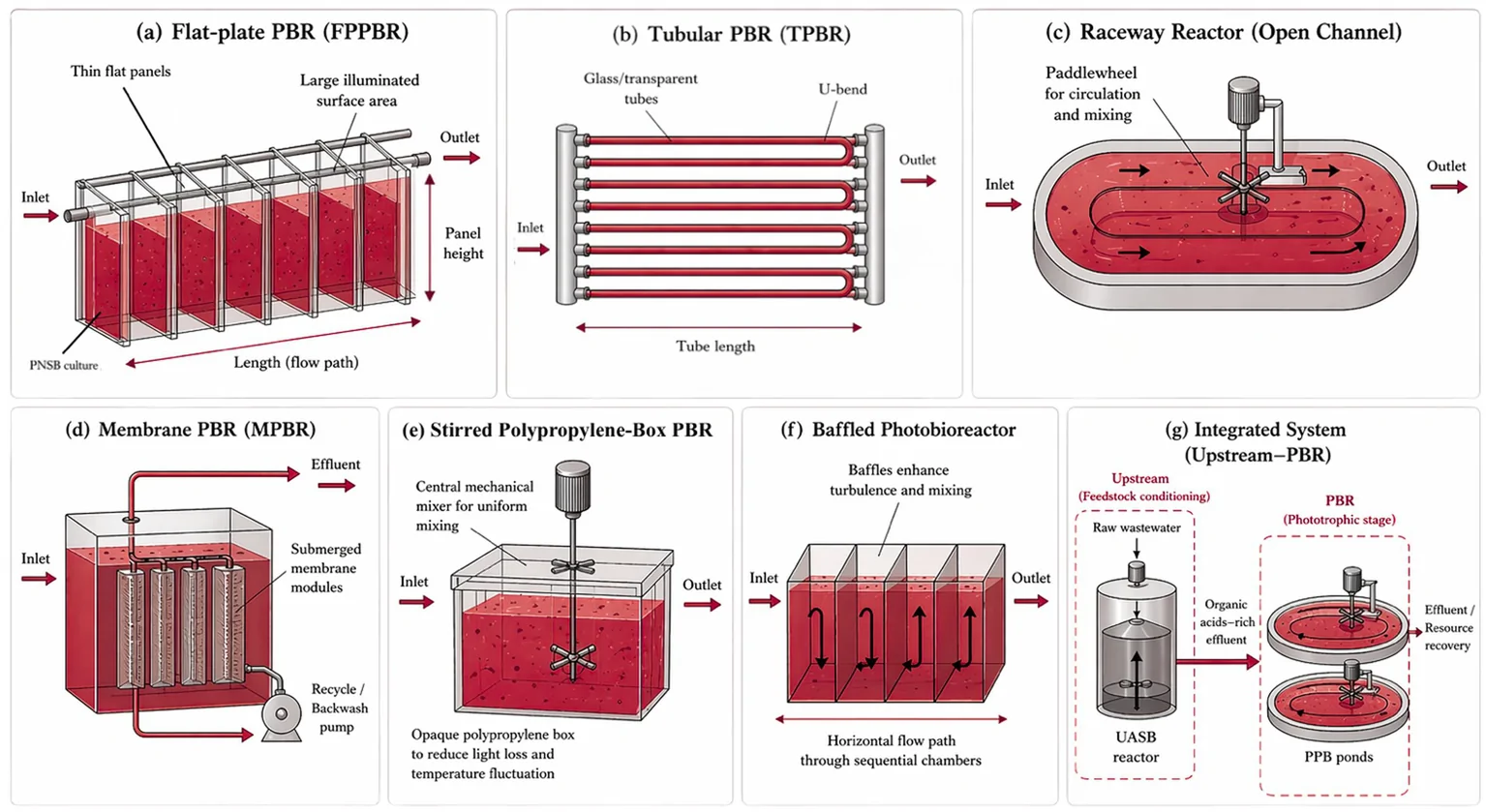
Schematic representation of major pilot-scale photobioreactor configurations used for PPB-based wastewater treatment and resource recovery: **(a)** flat-plate photobioreactor, **(b)** tubular photobioreactor, **(c)** raceway reactor, **(d)** membrane photobioreactor, **(e)** stirred polypropylene-box photobioreactor, **(f)** baffled photobioreactor, and **(g)** integrated UASB–PPB system.

The design and operational volume of photobioreactors, along with the mode of illumination, are among the most influential factors determining the performance of PPB in pilot-scale applications. Reactor geometry and scale directly affect light availability, biomass concentration, nutrient gradients, and mixing efficiency, parameters that are especially critical for photoheterotrophic organisms like PPB (Puyol et al., 2017a; Capson-Tojo et al., 2020; Hülsen et al., 2020). The reviewed pilot-scale studies encompass a diverse range of reactor configurations, with working volumes spanning from approximately 50 L to more than 9,600 L, reflecting the breadth of pilot-level scales currently being explored for outdoor phototrophic applications. A schematic comparison of the main photobioreactor configurations discussed in this section is presented in Figure 1 and their detailed geometric characteristics are provided in Supplementary Tables S3–S5.

### 5.1 Flat-plate photobioreactors (FPPBRs)

FPPBRs are among the most widely investigated reactor configurations for pilot-scale PPB cultivation because of their high illuminated surface area and efficient light utilization (Figure 1a). Their performance is primarily governed by reactor geometry, illumination, and biomass management.

Capson-Tojo et al. (2023b) operated a 953 L outdoor FPPBR treating pre-fermented poultry-processing wastewater under natural sunlight. The shallow optical path promoted uniform light distribution and stable PPB enrichment. However, progressive biofilm thickening caused self-shading, reducing photon penetration to deeper biofilm layers and limiting the fraction of metabolically active biomass. Although thicker biofilms improved biomass quality, periodic biofilm removal was required to restore light availability and maintain reactor productivity, highlighting biofilm management as a major engineering challenge during scale-up.

Hülsen et al. (2022a, 2022b) further demonstrated the potential of outdoor FPPBRs for treating poultry-processing, piggery, and chicken-processing wastewaters at working volumes ranging from 60 to 900 L. The studies showed that stable wastewater treatment and biomass production could be maintained across different reactor volumes, indicating that scale-up within this range did not compromise process performance. A notable finding was that selective near-infrared filtering enhanced PPB enrichment, particularly in nitrogen-rich wastewaters, highlighting light quality as an important operational variable for outdoor cultivation. In addition, biofilm-dominated growth produced protein-rich biomass suitable for single-cell protein applications. However, increasing biofilm accumulation reduced light penetration and intensified harvesting requirements, identifying biomass management as a key operational challenge during scale-up. The need for frequent biomass removal may also increase operational costs, indicating that harvesting strategies could become a major determinant of economic feasibility in larger systems (Hülsen et al., 2022a, 2022b; Capson-Tojo et al., 2023b; Shaikh et al., 2023c).

Gebicki et al. (2010) demonstrated stable hydrogen production in a 100 L outdoor flat-panel reactor using passive gas-driven mixing, indicating that mechanical agitation may not always be necessary at pilot-scale. However, process performance remained sensitive to light availability and mixing efficiency.

Overall, FPPBR performance depends more on light distribution, biomass retention, and harvesting efficiency than reactor volume. Their high illuminated surface area makes them attractive for wastewater treatment and SCP production under natural sunlight, although biofilm accumulation remains the principal operational challenge.

### 5.2 Tubular and horizontal photobioreactors

Pilot-scale tubular and horizontal tubular photobioreactors have primarily been investigated for hydrogen production and phototrophic biomass cultivation under controlled outdoor conditions. Their enclosed geometry provides a high illuminated surface area and enhanced control of culture conditions, but often introduces additional hydraulic, thermal, and operational complexity compared with simpler reactor configurations such as raceway ponds (Figure 1b).

Boran et al. (2010, 2012a, 2012b) demonstrated the feasibility of tubular photobioreactors for outdoor photofermentative hydrogen production using *Rhodobacter capsulatus*. Inclined tubes and supplemental illumination enhanced light availability, while internal cooling improved thermal regulation under high irradiance. However, hydrogen production remained sensitive to temperature fluctuations, and the use of polymeric tubing introduced gas permeability issues that required intermittent circulation to minimize hydrogen losses. These studies highlight that successful scale-up depends on balancing light utilization, temperature control, and gas retention.

Gebicki et al. (2010) further demonstrated stable hydrogen production under natural sunlight using a shallow tubular reactor. Although the horizontal configuration improved light penetration, active pumping was required to maintain circulation and mass transfer, increasing hydraulic complexity.

Carlozzi and Sacchi (2001) and Carlozzi et al. (2006) adopted a different strategy by submerging tubular photobioreactors in water, providing passive temperature regulation and reducing thermal fluctuations without active cooling. This approach improved operational stability while reducing cooling requirements.

Collectively, the reviewed studies indicate that tubular photobioreactors are particularly suitable for hydrogen production and other applications requiring controlled illumination and high light utilization. Their enclosed design supports efficient phototrophic activity, but often increases hydraulic complexity and sensitivity to temperature and gas management. Consequently, successful scale-up depends not only on reactor size but also on balancing light capture, circulation requirements, thermal regulation, and operational costs. From a reactor-selection perspective, tubular systems are most attractive for high-value products such as hydrogen, where productivity gains can justify the additional operational complexity.

### 5.3 Raceway reactors

Raceway reactors have been explored as a low-cost and operationally simple option for wastewater treatment and microbial protein production using PPB (Figure 1c). Doki et al. (2024) evaluated a 2.5 m^3^ pilot-scale raceway reactor treating fermented domestic wastewater under working volumes of 500–1,000 L. Carbon supplementation with acetate and propionate improved biomass production, protein content (40.3–43.9% w/w), and nutrient removal by overcoming carbon limitation. Although the shallow configuration enhanced light penetration, increasing biomass concentrations reduced the effective photic zone, while the open design increased susceptibility to microbial competition. Nevertheless, stable operation was maintained across different working volumes, indicating that raceway systems are a practical and low-cost option for wastewater treatment and SCP production when carbon availability and light limitation are properly managed.

### 5.4 Membrane photobioreactor

Membrane-assisted photobioreactors have been investigated as an alternative configuration to overcome biomass washout and effluent quality limitations commonly observed in suspended phototrophic systems (Figure 1d). Lu et al. (2019a) evaluated a pilot-scale membrane photobioreactor integrating phototrophic cultivation with submerged PVDF membrane modules. Membrane separation maintained high biomass concentrations by decoupling hydraulic and solids retention times, improving process stability and effluent quality. However, membrane fouling required regular cleaning, while membrane operation and artificial illumination increased energy demand. Consequently, membrane photobioreactors are best suited to applications requiring high biomass retention and high-quality effluents, provided that fouling and energy consumption can be effectively controlled.

### 5.5 Stirred polypropylene-box photobioreactor

Wada et al. (2023b) evaluated a stirred polypropylene-box photobioreactor for converting nitrogen-deficient fuel synthesis wastewater into protein-rich biomass (Figure 1e). The closed, mechanically mixed reactor supported PPB enrichment under nitrogen-limited conditions through biological nitrogen fixation, demonstrating simultaneous wastewater treatment and SCP production without external nitrogen supplementation. Light intensity strongly influenced biomass composition, with lower irradiance favoring protein accumulation and higher irradiance promoting pigment synthesis. Although organic matter removal exceeded 70%, nitrogen limitation slowed degradation, while biofilm accumulation reduced light penetration and increased harvesting requirements. These findings indicate that the stirred polypropylene-box photobioreactor is promising for nutrient-deficient industrial wastewaters but requires improved light management and energy-efficient operation for larger-scale implementation.

### 5.6 Baffled photobioreactor

Zhang et al. (2017) investigated pilot-scale photofermentative hydrogen production using a baffled photobioreactor composed of four sequential chambers with a total working volume of 4 m^3^ (Figure 1f). The compartmentalized design created substrate gradients and spatial separation of metabolic activity, while solar-fiber light delivery improved light distribution independently of reactor geometry. Passive circulation over internal baffles eliminated mechanical mixing but generated compartment-specific gradients in substrate concentration and metabolic activity. These results demonstrate that reactor hydrodynamics, light distribution, and hydraulic retention time collectively govern hydrogen production, making baffled photobioreactors attractive for hydrogen-oriented applications despite their greater design complexity (Zhang et al., 2017).

### 5.7 Phototrophic ponds coupled with UASB

Almeida et al. (2024) investigated an integrated pilot-scale configuration combining Upflow anaerobic sludge blanket (UASB) reactors with retrofitted PPB ponds to enable PHA production from domestic wastewater under outdoor conditions (Figure 1g). Coupling anaerobic fermentation with downstream phototrophic ponds enabled VFA production and subsequent PHA accumulation while allowing independent optimization of anaerobic and phototrophic processes. The system achieved PHA accumulation of up to 36% of volatile suspended solids, demonstrating the feasibility of outdoor PHA production at pilot-scale. However, seasonal temperature variation, fluctuating solar irradiance, incomplete upstream fermentation, and sludge accumulation reduced overall process performance, highlighting the importance of feedstock conditioning and stable fermentation for successful scale-up. Integrated UASB–PPB systems therefore represent a promising low-energy platform for PHA recovery from municipal and other readily fermentable wastewaters.

### 5.8 Comparative assessment of pilot-scale photobioreactor configurations

The reviewed pilot-scale photobioreactor configurations demonstrate that reactor geometry, illumination strategy, and biomass handling collectively determine system performance at scale, often more strongly than microbial capability alone. From a mechanistic perspective, reactor performance is fundamentally governed by light attenuation, which follows the Beer–Lambert law, where light intensity decreases exponentially with increasing optical path length (Capson-Tojo et al., 2022). Consequently, configurations with shorter light paths and higher surface-to-volume ratios provide improved photon availability and enhance photosynthetic efficiency (Grobbelaar et al., 1995; Zhang et al., 2001), whereas deeper systems experience significant light limitation, restricting active biomass zones and reducing overall productivity (Huesemann et al., 2013; Amini et al., 2025b). This establishes a direct relationship between reactor geometry and light utilization efficiency. To facilitate comparison among reactor configurations, key design and performance indicators, including light path, surface-to-volume ratio, reactor scale, and operational characteristics, are summarized in Supplementary Table S6.

Among the evaluated configurations, FPPBRs provided the most efficient light utilization and were particularly suited to SCP and PHA production, although biofilm accumulation reduced light penetration and increased harvesting requirements. Tubular and baffled photobioreactors offered greater process control and were more suitable for hydrogen production but required more complex hydraulic, thermal, and gas management. Raceway reactors represented the simplest and lowest-cost option for wastewater treatment and biomass production, although their performance was constrained by light limitation and carbon availability. Membrane photobioreactors achieved excellent biomass retention and effluent quality but were limited by membrane fouling and higher energy demand. The stirred polypropylene-box photobioreactor enabled controlled cultivation of PPB in nutrient-deficient industrial wastewaters but depended on artificial illumination, whereas integrated UASB–PPB systems supported low-energy PHA production provided that stable upstream fermentation and VFA generation were maintained.

Despite these differences, several engineering challenges were common across all reactor configurations. Light distribution remained the primary limitation as reactor size and biomass concentration increased, while biomass retention and harvesting became increasingly important during scale-up. Greater environmental control generally improved productivity but also increased operational complexity, energy demand, and maintenance requirements.

Successful scale-up therefore requires integrated optimization of reactor geometry, illumination, hydrodynamics, biomass management, and feedstock characteristics rather than improvement of a single design parameter. Reactor selection should ultimately be guided by the target resource recovery pathway, wastewater composition, and acceptable trade-offs between productivity, energy consumption, and operational complexity. Overall, the reviewed studies indicate that engineering constraints, rather than microbial limitations alone, become the dominant factors governing successful pilot-scale implementation of PPB-based resource recovery systems.

## 6 Microbial community dynamics

Microbial community composition is a key determinant of performance and stability in pilot-scale phototrophic wastewater treatment systems. Evidence from recent pilot-scale studies indicates that microbial community structure is primarily shaped by reactor configuration and operating conditions, while wastewater type influences enrichment patterns within these engineered environments. Rather than being a passive outcome of operation, microbial organization actively governs substrate conversion pathways, biomass quality, and resilience under outdoor conditions.

In outdoor FPPBRs treating piggery wastewater and chicken-processing wastewater, microbial community analyses consistently showed strong enrichment of PPB, particularly within attached biofilms. In PWW-fed systems, *Rhodopseudomonas* spp. accounted for up to 57% of the biofilm community, with *Blastochloris* spp. contributing up to 11%, reflecting their ability to tolerate temperature variability and persist under long solid retention times (Hülsen et al., 2022a). This pattern suggests that biofilm-based operation acts as a selective ecological niche, favoring phototrophic taxa adapted to fluctuating outdoor conditions. In chicken processing wastewater (CWW)-fed FPPBRs, *Rhodobacter* spp. (27%) and *Rhodopseudomonas* spp. (28%) were dominant, while total PPB abundance ranged from 15 to 63% depending on reactor volume (Hülsen et al., 2022a). The observed variation across volumes indicates that hydrodynamics and available attachment area influence not only biomass productivity but also microbial partitioning within the system.

Alongside PPB, fermentative and acidogenic microorganisms were consistently detected and contributed to VFA generation, establishing a metabolic coupling in which PPB relied on upstream fermentation rather than direct utilization of complex organics (Hülsen et al., 2022a). The interaction between PPB and auxiliary microbial populations plays a critical role in sustaining process performance. Fermentative bacteria convert complex organic matter into volatile fatty acids (VFAs), which serve as preferred substrates for PPB under photoheterotrophic conditions, while sulfur- and nitrogen-cycling microorganisms contribute to nutrient transformation and redox balance (Lazaro et al., 2012; Cerruti et al., 2023). These interactions indicate that pilot-scale PPB systems function as structured microbial consortia rather than isolated phototrophic cultures. Functionally, this microbial organization enhances substrate conversion efficiency by coupling upstream fermentation with downstream photoheterotrophic metabolism, thereby improving carbon utilization, nutrient transformation, and overall process stability. These findings suggest that maintaining functional interactions among microbial guilds is as important as enriching PPB abundance for sustained pilot-scale performance.

Light regime further influenced microbial community composition in FPPBRs. In CWW systems, shifting from near-infrared-filtered illumination to full-spectrum sunlight reduced PPB abundance, likely due to increased competition from microalgae and aerobic heterotrophs (Hülsen et al., 2022b). This demonstrates that illumination acts not only as an energy input but also as a selective pressure shaping microbial structure. The use of IR/NIR-selective illumination acts as a targeted ecological selection pressure that promotes PPB dominance over competing phototrophs such as microalgae (Nagashima et al., 2021; Yu et al., 2022; Capson-Tojo et al., 2023a). Although functional gene-level analyses are rarely reported in pilot-scale studies, these mechanisms likely underpin the observed enrichment patterns. For example, genes involved in PHA biosynthesis (phaC), nitrogen fixation (nifH), sulfur metabolism, and photosynthetic energy conversion would provide mechanistic insight into how operational conditions regulate resource recovery and microbial adaptation under pilot-scale conditions. In contrast to FPPBRs, raceway systems supported more diverse and less selective microbial communities. In a two-stage photoheterotrophic raceway treating domestic sewage, phototrophic enrichment depended on upstream anaerobic fermentation, and the microbial community consisted of PPB together with microorganisms involved in nitrogen fixation, sulfur cycling, and organic matter degradation (Doki et al., 2024). Compared with FPPBRs, raceway systems exhibited lower PPB selectivity and stronger dependence on substrate composition and feeding strategy, reflecting weaker physical and optical control. Collectively, these findings indicate that reactor configuration influences microbial community structure primarily through its effects on light availability, biomass retention, oxygen exposure, and substrate conversion pathways.

Across the reviewed pilot-scale studies, *Rhodopseudomonas*, *Rhodobacter*, and *Blastochloris* consistently emerged as dominant PPB genera. Community stability was strongly influenced by operational factors including light regime, oxygen availability, biomass retention strategy, and substrate composition. Although temporary losses of PPB dominance occurred under unfavorable conditions, re-establishment of selective pressures generally enabled community recovery, indicating a degree of ecological resilience at pilot-scale. These observations suggest that operational conditions exert stronger control over community structure than wastewater type alone. Despite recent advances, microbial ecology remains one of the least characterized aspects of pilot-scale PPB systems. Quantitative relationships between operating conditions and community structure remain poorly understood, and advanced approaches such as co-occurrence network analysis, metabolomics, and functional gene characterization have rarely been applied. As a result, the mechanisms linking microbial interactions to process performance are still largely inferred rather than directly demonstrated. Future research should place greater emphasis on linking microbial community function with reactor operation, biomass harvesting, and process performance through integrated omics, metabolic pathway analysis, and process-level measurements rather than relying solely on taxonomic characterization. In particular, combining sequencing approaches with functional gene analysis (e.g., *phaC* for PHA synthesis and *nifH* for nitrogen fixation), metabolomics, and multivariate statistical tools could help establish quantitative links between reactor design, microbial interactions, and resource recovery outcomes, ultimately enabling more reliable scale-up and operation of PPB-based resource recovery systems.

## 7 Resource recovery strategies and recovered products

Pilot-scale resource recovery outcomes are summarized in this section, with quantitative yields and biomass compositions presented in Table 2. As shown in Table 2, product quantification methods vary considerably across studies (e.g., crude protein estimated as N × 6.25 versus direct protein assays such as Lowry), which introduces variability in reported values and limits direct comparability. The reported products span bulk biomass and intracellular metabolites, reflecting how reactor design, growth mode, and operating conditions shape valorization pathways in PPB-based systems. Despite the compilation of yield data, cross-study normalization remains challenging due to differences in analytical methods, reporting units, and biomass bases. Consequently, efficiency differences across studies are interpreted qualitatively, with variations in product yields primarily governed by process conditions such as light availability, nutrient balance, reactor configuration, and growth mode, which regulate carbon flux distribution within PPB metabolism.

**Table 2:** Resource recovery yields and productivities achieved in pilot-scale phototrophic systems using different wastewater and synthetic feedstocks.

Study	Feedstock/Medium	Resources recovered	Reported yield or productivity	Measurement method
Almeida et al. (2024)	Domestic wastewater	PHA	36% (g PHA/g VSS)	PHA: Gas Chromatography
Doki et al. (2024)	Sewage	SCP and lipids	SCP 43.9%; and lipids 25.5%	SCP: modified Lowry assay; lipids: gravimetric;
Wada et al. (2023b)	Fuel synthesis wastewater	SCP, Crts, BChls, and lipids	SCP 43.4%; lipids 25.3%; Crts 25 mg.g^-1^biomass; BChls 24.9 mg.g^-1^biomass	SCP: modified Lowry assay; lipids: NR; Crts/BChls: spectrophotometric method
Capson-Tojo et al. (2023b)	Poultry processing wastewater	SCP	SCP 57.5%	SCP: crude protein (N × 6.25)
Hülsen et al. (2022b)	Poultry processing wastewater	SCP	SCP 58%	SCP: crude protein (N × 6.25)
Hülsen et al. (2022a)	Piggery and Chicken processing wastewater	SCP, Crts, and BChls	SCP 50–65%; Crts 5–9 mg.g^-1^VS; BChls 11–20 mg.g^-1^VS	SCP: crude protein (N × 6.25)
Lu et al. (2019a)	Brewery wastewater	SCP, Crts, BChls, polysaccharides, and coenzyme Q10	SCP 42.1%; polysaccharides 17.8%; Crts 0.25%; BChls 1.1%; and coenzyme Q10 3.7%	SCP: modified Lowry assay; polysaccharides: phenol-sulfuric acid method; Crts and BChls: spectrophotometric method; coenzyme Q10: HPLC
Zhang et al. (2017)	Synthetic medium (glucose, 10 g/L)	H_2_	234–381 mol H₂ d^-1^	H_2_: Gas Chromatography
Adessi et al. (2012)	RPP medium (malate 4 g/L, glutamate 1 g/L)	H_2_	60.3 L H_2_ over the production period	H_2_: Gas Chromatography and Amperometric micro-sensor
Boran et al. (2012a)	MBP medium (with acetate 20 mM)	H_2_	0.35 mol H_2_ mol^-1^ acetic acid fed	H_2_: Gas Chromatography
Boran et al. (2012b)	Thick juice dark-fermenter effluent	H_2_	0.40 mol H_2_ mol^-1^ acetic acid consumed	H_2_: Gas Chromatography
Boran et al. (2010)	MBP medium (with acetate 40 mM)	H_2_	0.60 mol H₂ mol⁻¹ acetic acid fed	H_2_: Gas Chromatography
Gebicki et al. (2010)	Synthetic organic acids (acetic acid + sodium lactate)	H_2_	3,350 – 3,690 mL H₂ m^-2^ d^-1^	H_2_: Gas Chromatography
Carlozzi and Sacchi (2001)	Modified van Niel synthetic medium (sodium acetate, ammonium chloride, and yeast extract)	PHB, SCP, Crts, and BChls	PHB 1–4%; SCP 51–61%; Crts 2–4 mg.g^-1^ DW; and BChls 10–21 mg.g^-1^ DW	PHB: spectrophotometric method; SCP: Lowry protein method; Crts and BChls: spectrophotometric method

SCP, single-cell protein; PHA, polyhydroxyalkanoates; PHB, polyhydroxybutyrate; Crts, carotenoids; BChls, bacteriochlorophylls; VSS, volatile suspended solids; VS, volatile solids; d.w., dry weight; NR, Nile Red fluorescence assay; HPLC, high-performance liquid chromatography; H_2_, hydrogen; MBP, modified Biebl and Pfennig medium; RPP, *Rhodopseudomonas palustris* producing growth medium.

### 7.1 Single-cell protein

SCP is the most technologically mature and consistently reported PPB-derived product at pilot-scale. Across the reviewed studies, protein contents ranged from 42 to 65% of dry biomass, demonstrating that high-quality microbial protein can be produced from diverse wastewaters, including poultry-processing (Hülsen et al., 2022b), piggery (Hülsen et al., 2022a), brewery (Lu et al., 2019a), and fuel-synthesis wastewaters (Wada et al., 2023b). However, protein yield depended more strongly on biomass retention strategy, nitrogen availability, light regime, and analytical methodology than on wastewater type.

Biofilm-based FPPBRs achieved the highest protein contents (50–65% crude protein) when treating poultry-processing and piggery wastewaters (Hülsen et al., 2022a, 2022b; Capson-Tojo et al., 2023b). In addition to higher protein concentrations, attached biomass exhibited lower ash content and improved settleability than suspended biomass, making it more suitable for feed applications. These findings indicate that attached-growth operation enhances both biomass quality and PPB enrichment, although excessive biofilm accumulation reduces light penetration and reactor productivity.

Comparable protein contents (56–61% dry biomass) were reported for *Rhodopseudomonas palustris* cultivated in outdoor tubular photobioreactors (Carlozzi and Sacchi, 2001). Unlike biofilm systems, these reactors maintained SCP production despite natural environmental fluctuations, suggesting that metabolic flexibility and controlled cultivation conditions can sustain protein synthesis during outdoor operation.

Lower but consistent protein contents (42–44%) were reported for stirred polypropylene-box photobioreactor treating fuel-synthesis wastewater (Wada et al., 2023b), raceway reactors treating domestic sewage (Doki et al., 2024), and membrane photobioreactors treating brewery wastewater (Lu et al., 2019a). Although these values were approximately 20–30% lower than those achieved in FPPBRs, stable SCP production was maintained across different wastewater types and reactor configurations. In particular, continuous membrane reactor operation for 440 days demonstrated the long-term stability of PPB-based SCP production under pilot-scale conditions.

Direct comparison of SCP yields across studies should be interpreted cautiously because protein quantification methods vary substantially. Several studies (Hülsen et al., 2022a, 2022b; Capson-Tojo et al., 2023b) estimated crude protein using the conventional nitrogen-to-protein conversion factor (N × 6.25), whereas others (Carlozzi and Sacchi, 2001; Lu et al., 2019a; Wada et al., 2023b; Doki et al., 2024) employed direct protein assays such as the Lowry method. Since crude protein calculations include non-protein nitrogen fractions, reported values may partly reflect analytical methodology rather than true biological variation.

Overall, the available evidence indicates that biomass retention strategy and light management exert stronger influences on SCP yield than wastewater composition. Biofilm-based FPPBRs and tubular reactors consistently produced the highest protein contents, whereas raceway, membrane, and stirred polypropylene-box photobioreactors provided slightly lower but stable yields. These findings identify SCP as the most promising near-term commercialization pathway for PPB-based wastewater biorefineries because of its consistently high protein content, broad wastewater compatibility, and established demand in aquaculture and animal feed markets.

### 7.2 Hydrogen gas

Hydrogen production represents a distinct PPB-based resource recovery pathway that differs fundamentally from biomass-oriented applications. Unlike SCP production, hydrogen generation is typically achieved under growth-limiting conditions where electrons are redirected from biomass synthesis toward photofermentative hydrogen evolution. Consequently, reactor design, light utilization, gas management, and maintenance of strict anaerobic conditions become the primary determinants of process performance (Boran et al., 2010, 2012a, 2012b; Gebicki et al., 2010; Adessi et al., 2012; Zhang et al., 2017). Across the reviewed pilot-scale studies, hydrogen production was investigated predominantly in closed tubular, flat-panel, and baffled photobioreactors operated with pure cultures, most commonly *Rhodobacter capsulatus.*

Tubular photobioreactors remain the most extensively studied configuration for pilot-scale hydrogen production. Adessi et al. (2012) demonstrated sustained outdoor hydrogen generation in a 50 L tubular reactor, while Boran et al. (2010, 2012a, 2012b) reported hydrogen yields of 0.35–0.60 mol H₂ mol⁻¹ acetic acid in solar tubular photobioreactors, with the yield basis defined as acetic acid fed or consumed depending on the study. Reactor performance was strongly influenced by light availability, substrate concentration, temperature control, and maintenance of low hydrogen partial pressure. The findings highlight the importance of balancing light capture and gas removal, as hydrogen accumulation can inhibit nitrogenase activity and reduce overall productivity.

Direct comparison of reactor configurations demonstrated the importance of reactor geometry. Under comparable operating conditions, flat-plate photobioreactors achieved a higher areal hydrogen productivity (3,690 mL H₂ m^-2^ d^-1^) than tubular reactors (3,350 mL H₂ m^-2^ d^-1^), representing an improvement of approximately 10%, primarily because of shorter optical paths, improved light distribution, and more efficient gas disengagement (Gebicki et al., 2010). At a larger scale, a 4 m^3^ baffled photobioreactor sustained hydrogen production rates of 234–381 mol H₂ d^-1^, demonstrating that compartmentalized reactor design can maintain continuous hydrogen recovery by improving spatial organization of metabolic activity (Zhang et al., 2017).

Collectively, the reviewed studies indicate that hydrogen production remains one of the most technically demanding PPB-based resource recovery pathways. Compared with SCP production, hydrogen systems require greater control over illumination, gas management, temperature, and oxygen exposure. While flat-panel and tubular reactors generally achieve the highest hydrogen productivities, large-scale implementation is constrained by light attenuation, hydrogen inhibition, gas leakage, and the continued reliance on pure cultures and synthetic substrates. These challenges explain why hydrogen recovery remains less mature than SCP production at pilot-scale despite its demonstrated technical feasibility. Future development should focus on improving light delivery, continuous hydrogen removal, and operation with mixed cultures and real wastewaters to enhance process robustness and economic viability.

### 7.3 Polyhydroxyalkanoates

PHAs, particularly PHB, represent one of the most attractive intracellular products recovered from PPB due to their potential application as biodegradable bioplastics. Unlike SCP, which is produced under a broad range of operating conditions, PHA accumulation occurs only when carbon availability, nutrient balance, and environmental conditions favor diversion of cellular carbon flux toward intracellular storage. Consequently, PHA production is generally more sensitive to reactor operation and process control than biomass production.

Pilot-scale studies demonstrate considerable variation in PHA accumulation depending on reactor configuration and operating strategy. The highest accumulation was reported in an integrated UASB–PPB system treating molasses-supplemented domestic wastewater, where mixed PPB cultures accumulated up to 36% of volatile suspended solids under feast–famine operation (Almeida et al., 2024).

In contrast, an outdoor tubular photobioreactor cultivating *Rhodopseudomonas palustris* achieved approximately 4% PHB (dry biomass) under continuous cultivation (Carlozzi and Sacchi, 2001). This approximately nine-fold difference indicates that feast–famine operation, VFA-rich feedstocks, and controlled nutrient limitation promote substantially greater polymer storage than conventional continuous cultivation.

Across the reviewed studies, PHA accumulation was governed primarily by carbon availability, nutrient imbalance, light regime, and reactor operation. Carbon-rich conditions and higher irradiance generally promoted polymer storage, whereas nutrient-replete or light-limited conditions favored biomass growth and intracellular PHB consumption. Compared with SCP production, PHA recovery remains less frequently reported at pilot-scale and requires tighter process control. Nevertheless, the high accumulation achieved in integrated UASB–PPB systems demonstrates that mixed-culture PPB can convert wastewater-derived VFAs into bioplastic precursors under realistic outdoor conditions, highlighting PHA production as one of the most promising high-value resource recovery pathways for future PPB biorefineries.

### 7.4 Pigments (Crts and BChls)

Photosynthetic pigments, particularly Crts and BChls, are intrinsic components of PPB biomass and are routinely recovered alongside proteins and storage polymers. Unlike SCP or PHAs, pigment production does not generally require specific process modifications, as these compounds are directly linked to the photosynthetic machinery of PPB. Consequently, pigment accumulation is primarily influenced by light availability, reactor configuration, growth mode, and environmental conditions.

Pilot-scale studies demonstrated consistent pigment production across multiple reactor configurations, although concentrations varied considerably. Biofilm-based flat-plate photobioreactors achieved 8.7 mg Crts g^-1^ VS and 19.5 mg BChls g^-1^ VS, with attached biomass containing higher pigment concentrations than suspended biomass (Hülsen et al., 2022a). Similar pigment levels were maintained during 440 days of continuous operation in membrane photobioreactors treating brewery wastewater (Lu et al., 2019a), indicating long-term process stability. The highest pigment concentrations were reported in a stirred polypropylene-box photobioreactor treating nitrogen-deficient fuel-synthesis wastewater, reaching approximately 25 mg Crts g^-1^ dry biomass and 24.9 mg BChls g^-1^ dry biomass under controlled illumination (Wada et al., 2023b), representing approximately a three-fold increase in carotenoid content and a 28% increase in BChls content compared with biofilm-based FPPBRs. Outdoor tubular photobioreactors also exhibited seasonal increases in both pigments during periods of higher solar irradiance (Carlozzi and Sacchi, 2001), further demonstrating the strong influence of light availability on pigment biosynthesis.

Across the reviewed studies, higher irradiance and attached-growth operation consistently promoted pigment accumulation, reflecting increased investment in light harvesting and photoprotection under enhanced photosynthetic activity. Compared with SCP and PHAs, pigments are recovered as co-products rather than primary target products, providing an additional valorization pathway with minimal modification of reactor operation. Future research should focus on optimizing illumination strategies and downstream extraction processes to maximize pigment recovery while maintaining wastewater treatment performance.

### 7.5 Lipids

Lipids represent a secondary but consistently reported component of PPB biomass in pilot-scale systems. Unlike PHAs, lipid accumulation was not intentionally promoted through nutrient limitation or specialized operating strategies, but instead occurred as part of normal photoheterotrophic growth under carbon-rich conditions. Consequently, lipids were generally recovered together with SCP, pigments, and other biomass constituents rather than as a primary target product. Across the reviewed pilot-scale studies, lipid contents remained remarkably consistent (25.3–25.5% dry biomass) despite differences in wastewater type and reactor configuration. Wada et al. (2023b) reported 25.3% lipid content in PPB biomass in a stirred polypropylene-box photobioreactor treating fuel-synthesis wastewater, while Doki et al. (2024) reported a comparable 25.5% in a two-stage anaerobic fermentation-raceway system treating domestic sewage. The difference between the two systems was less than 1%, suggesting that lipid synthesis is a relatively stable metabolic characteristic of PPB under continuous cultivation when sufficient organic carbon is available. Compared with SCP and PHAs, lipids have received limited attention as a dedicated resource recovery target in pilot-scale PPB systems. However, their consistent accumulation at approximately one-quarter of biomass dry weight highlights an additional valorization opportunity. Future studies should evaluate whether operational strategies commonly used to enhance lipid accumulation in other microbial systems, such as nutrient limitation or carbon excess, can further increase lipid yields while maintaining wastewater treatment performance.

### 7.6 Polysaccharides and coenzyme Q10

Polysaccharides and coenzyme Q10 are among the least investigated PPB-derived products at pilot-scale and have been reported only in the membrane-assisted photobioreactor study of Lu et al. (2019a). In this membrane photobioreactor, polysaccharides accounted for 17.8% of biomass dry weight, while CoQ10 reached 38.6 mg g^-1^ dry biomass during continuous treatment of brewery wastewater. Both compounds were quantified after 440 days of continuous operation, demonstrating sustained production under photo-microaerobic conditions. In contrast to pigments, which have been consistently reported across multiple pilot-scale reactor configurations, polysaccharides and CoQ10 have been quantified in only one pilot-scale study, highlighting a significant knowledge gap despite their apparent commercial potential. Despite the limited evidence available, these results demonstrate that PPB biomass can simultaneously produce additional high-value compounds alongside SCP, PHAs, pigments, and lipids. In particular, CoQ10 is a commercially relevant bioactive molecule with applications in nutraceutical, pharmaceutical, and cosmetic industries. The simultaneous recovery of these products during wastewater treatment further supports the development of PPB-based multiproduct biorefineries, improving the potential economic value of the process. However, additional pilot-scale studies are required to determine whether polysaccharide and CoQ10 production can be reproducibly achieved across different reactor configurations, wastewater types, and operating conditions, and whether their recovery is economically feasible.

### 7.7 End-use applications and commercial outlook

Although pilot-scale studies have demonstrated the technical feasibility of recovering a diverse range of PPB-derived products, successful industrial deployment ultimately depends on their commercial value, market demand, scalability, and regulatory acceptance rather than production alone. The recovered products differ considerably in market maturity, end-use sectors, and technology readiness, ranging from bulk commodities such as SCP and biodegradable plastics to high-value specialty chemicals including carotenoids, coenzyme Q10, and 5-aminolevulinic acid. This section critically evaluates the principal commercial opportunities and remaining barriers that will determine the transition of PPB-based wastewater biorefineries from pilot-scale demonstrations to industrial implementation (Chamodi et al., 2025; Morrison and Bose, 2025).

Among PPB-derived products, SCP currently represents the most commercially advanced opportunity. Increasing global protein demand, projected to rise by approximately 40% to nearly double by 2050, together with growing concerns regarding food security, land scarcity, and environmental sustainability, is accelerating the transition toward microbial proteins as alternative protein sources (Ye et al., 2024; Ayele et al., 2026). The global SCP market was valued at USD 10.36 billion in 2023 and is expected to reach USD 22.49 billion by 2032, while another market assessment projects growth from USD 13.1 billion in 2022 to USD 74.6 billion by 2032, reflecting strong commercial momentum (Ye et al., 2024; Chamodi et al., 2025). PPB biomass typically contains 60–72% crude protein with a balanced amino acid profile comparable to soybean and is enriched with vitamins, carotenoids, lipids, minerals, nucleotides, and other bioactive compounds, making it particularly attractive for aquaculture, livestock, poultry, pet food, and human nutrition (Chamodi et al., 2025; Morrison and Bose, 2025). The commercial feasibility of microbial proteins is demonstrated by products such as FeedKind® Aqua, KnipBio Meal, Novacq™, UniProtein®, CeFi® Pro, Solein®, NovoMeal, Pruteen, and Quorn™, with approximately 35 companies already producing SCP commercially (Ye et al., 2024; Chamodi et al., 2025). Nevertheless, broader commercialization remains constrained by production costs, harvesting efficiency, downstream processing, nucleic acid reduction, digestibility, regulatory approval, product consistency, and consumer acceptance, emphasizing the need for improved bioreactor design, process optimization, and integrated biorefinery approaches (Ye et al., 2024; Chamodi et al., 2025).

Polyhydroxyalkanoates (PHAs) represent another commercially attractive product because they provide biodegradable and biocompatible alternatives to petroleum-based plastics. Their principal end-user industries include food packaging, agriculture, disposable consumer products, automotive components, textiles, electronics, paper products, biomedical devices, tissue engineering, drug delivery systems, vascular grafts, regenerative medicine, and 3D printing (Zhang Z. et al., 2024; Jayalath and de Alwis, 2025). Industrial production has already reached commercial maturity in several regions, with manufacturing capacities approaching 50,000 t year^-1^, demonstrating increasing market acceptance (Jayalath and de Alwis, 2025). For PPB, wastewater-derived PHA production provides an additional economic advantage by simultaneously achieving wastewater treatment and bioplastic synthesis while reducing dependence on refined carbon substrates (Morrison and Bose, 2025). However, commercialization remains limited by relatively low intracellular PHA accumulation, high downstream processing costs, and production costs of approximately US$4–6 kg^-1^, compared with US$1–2 kg^-1^ for conventional plastics (Gundlapalli and Ganesan, 2025). Continued advances in low-cost waste feedstocks, microbial engineering, continuous fermentation, AI-assisted process optimization, and sustainable extraction technologies are expected to improve the economic competitiveness of PHA production (Zhang Z. et al., 2024; Gundlapalli and Ganesan, 2025).

In addition to proteins and PHAs, PPB synthesize several high-value specialty chemicals that substantially improve the economics of integrated biorefineries. Carotenoids have established applications in the aquaculture, food, nutraceutical, pharmaceutical, cosmetic, and functional food industries, where they function as antioxidants, vitamin A precursors, and natural pigments (de Carvalho and Caramujo, 2017). The global carotenoid market exceeded US$1.5 billion and continues to expand with increasing demand for natural bioactive compounds and aquaculture products (de Carvalho and Caramujo, 2017). In shrimp aquaculture, carotenoids improve pigmentation, growth, antioxidant capacity, immune function, and product quality, thereby directly enhancing market value and consumer acceptance (Liu et al., 2025). Likewise, Coenzyme Q10 is one of the highest-value metabolites produced by PPB, serving the pharmaceutical, nutraceutical, cosmetic, food, and healthcare industries. Global demand is estimated at 988–1369 tonnes annually, with a market exceeding USD 890 million, while PPB-based production has already been demonstrated at 150-L pilot and 80,000-L industrial scales, highlighting its commercial scalability (He et al., 2021).

Another promising PPB-derived specialty product is 5-ALA, which has rapidly expanding applications in medicine, agriculture, livestock, and aquaculture. In medicine, 5-ALA is extensively used for photodynamic diagnosis and photodynamic therapy, whereas in agriculture it functions as a plant growth regulator that enhances crop productivity and abiotic stress tolerance. It is also increasingly utilized as a feed additive to improve growth performance and immune responses in livestock and aquatic animals (Zhou et al., 2024). The global 5-ALA market is projected to reach approximately US$159.58 million by 2030, while recent metabolic engineering has achieved record microbial production of 63.39 g L^-1^ with an estimated Technology Readiness Level (TRL) 6, demonstrating strong commercial readiness for industrial biomanufacturing (Zhou et al., 2024).

Beyond proteins and specialty chemicals, PPB offer opportunities for producing microbial lipids, biohydrogen, and biofertilizers, further strengthening the economic attractiveness of wastewater biorefineries. Waste-derived microbial lipids are suitable for biodiesel, oleochemicals, food ingredients, animal feed, cosmetics, and nutraceuticals, while co-production with extracellular biopolymers further improves process profitability (Byrtusová et al., 2025). Biohydrogen is emerging as an important renewable fuel for the transportation, industrial, agricultural, and decentralized energy sectors, with hydrogen demand projected to increase substantially under global decarbonization strategies. However, commercialization remains limited by feedstock logistics, infrastructure, biological productivity, and scale-up challenges (Zahoor et al., 2025). In agriculture, PPB biomass also functions as a biofertilizer and plant biostimulant, enhancing nitrogen fixation, phosphorus recovery, crop productivity, and fertilizer-use efficiency, thereby supporting sustainable agricultural production while creating additional value from wastewater treatment (Morrison and Bose, 2025).

Overall, the commercial potential of PPB technologies lies in their ability to integrate wastewater treatment with the recovery of multiple value-added products for diverse industrial sectors. However, successful commercialization will ultimately depend on achieving an optimal balance between product value, process efficiency, downstream recovery, and economic viability, rather than maximizing individual product yields alone.

## 8 Critical evaluation and research gaps: operational constraints and scale-up implications

Pilot-scale phototrophic systems based on PPB have demonstrated clear potential for simultaneous wastewater treatment and resource recovery. However, results across studies also reveal recurring operational constraints that limit process robustness, cross-study comparability, and scalability. These challenges arise primarily from biomass handling, light delivery, reactor configuration, substrate composition, and cost-sensitive operational choices, rather than from intrinsic biological limitations of PPB. Importantly, many of these constraints emerge or intensify with increasing system scale, underscoring the need to distinguish pilot-scale-specific mechanisms from laboratory-scale observations. Addressing these issues is essential for translating pilot-scale success into industrially viable systems. Although individual studies identify key operational bottlenecks, direct quantitative comparisons of their relative impacts across reactor configurations remain scarce.

### 8.1 What fundamentally changes at pilot-scale

Laboratory-scale PPB systems typically operate under highly controlled conditions characterized by short optical path lengths, uniform illumination, efficient mixing, stable temperatures, and relatively constant wastewater composition (Molina Grima et al., 2000; Capson-Tojo et al., 2022). Under these conditions, reactor performance is primarily governed by microbial metabolism and process optimization. However, when these systems are translated to pilot-scale, several engineering and operational constraints emerge that cannot be predicted through simple volumetric scaling alone (Molina Grima et al., 2000; De Vree et al., 2015). Consequently, pilot-scale operation represents a critical transition stage where biological performance becomes increasingly influenced by reactor design, environmental variability, and operational practicality.

Among these constraints, light transfer becomes the dominant engineering challenge. Increasing reactor volume generally reduces the surface-to-volume ratio and increases optical path length, creating strong light gradients throughout the culture (Janssen et al., 2003). As biomass concentration increases, self-shading and biofilm formation further restrict photon penetration, reducing the fraction of metabolically active biomass. Across the reviewed pilot-scale studies, light limitation emerged as a recurring bottleneck regardless of reactor configuration, influencing microbial community structure, biomass productivity, nutrient removal efficiency, and product formation (Hülsen et al., 2022a; Capson-Tojo et al., 2023b). These observations indicate that successful scale-up depends more on effective light distribution than on reactor volume alone.

Scale-up also alters mixing and mass-transfer dynamics. Laboratory systems generally maintain near-homogeneous conditions, whereas pilot-scale reactors develop gradients in substrate concentration, dissolved gasses, temperature, and redox conditions (Molina Grima et al., 2000; Androga et al., 2012). Such gradients become particularly evident in elongated tubular reactors, compartmentalized systems, and large outdoor ponds, where local environmental conditions may vary considerably within the same reactor. As a result, reactor hydrodynamics increasingly influence process performance, product formation, and microbial stability as scale increases.

Another important change is the transition from controlled laboratory cultures to complex mixed microbial communities. Under non-sterile pilot-scale operation, microbial selection is driven by environmental pressures such as light regime, oxygen availability, nutrient availability, and biomass retention strategy (Hülsen et al., 2022a; Capson-Tojo et al., 2023b). Consequently, system performance depends not only on the characteristics of individual PNSB species but also on interactions among phototrophic, fermentative, sulfur-cycling, and nitrogen-cycling microorganisms. Maintaining stable community structure therefore becomes an operational challenge that is rarely encountered under laboratory conditions.

Pilot-scale systems are also exposed to temporal variability that is often absent in laboratory studies. Fluctuations in solar irradiance, temperature, seasonal conditions, and wastewater composition introduce operational uncertainty and can significantly influence treatment efficiency and product yields (Hülsen et al., 2022a, 2022b; Capson-Tojo et al., 2023b). Unlike laboratory experiments conducted under constant conditions, pilot-scale systems must demonstrate resilience to these variations while maintaining stable performance over extended periods.

Another important challenge that becomes increasingly significant with scale is feedstock conditioning and nutrient management. Many wastewaters do not naturally possess the nutrient balance required for optimal PPB growth and product formation. As demonstrated in several reviewed studies, wastewater fermentation, pH adjustment, volatile fatty acid generation, and supplementation with minerals, vitamins, trace elements, or additional nutrient sources were often required to sustain process performance (Wada et al., 2023b; Almeida et al., 2024; Doki et al., 2024). While such interventions are feasible at pilot-scale, the procurement, storage, transportation, and dosing of chemicals can become major economic and logistical challenges at industrial scales. Consequently, wastewater suitability should be evaluated not only according to treatment performance but also according to the extent of conditioning required to support PPB metabolism.

Finally, resource recovery introduces challenges that extend beyond reactor operation itself. As biomass production increases, harvesting, dewatering, drying, storage, and transportation become increasingly important determinants of overall process feasibility. Unlike laboratory studies, where biomass handling is often performed manually and at small scale, pilot-scale systems reveal the significant operational and economic burden associated with downstream processing. These requirements can strongly influence the viability of recovered products, particularly for low-value commodities such as bulk biomass and biofertilizers. Collectively, these observations demonstrate that the primary barriers to scale-up are not biological limitations of PPB, but rather engineering, operational, and economic constraints associated with light delivery, mass transfer, microbial stability, feedstock conditioning, and downstream biomass management. Understanding how these factors interact at pilot-scale is essential for developing robust and commercially viable PPB-based wastewater resource recovery systems.

### 8.2 Biomass harvesting and product separation

Biomass harvesting remains one of the most significant barriers to the large-scale implementation of PPB-based resource recovery systems. Although most pilot-scale studies focus on wastewater treatment and product formation, comparatively little attention has been given to harvesting efficiency, energy demand, and downstream processing requirements. Harvesting challenges vary according to reactor configuration. Biofilm-based flat-plate photobioreactors produce high-quality protein-rich biomass but require periodic scraping or mechanical removal of attached biomass, increasing labor and operational complexity (Hülsen et al., 2022a, 2022b; Capson-Tojo et al., 2023b). In contrast, suspended-growth systems such as raceway ponds and stirred reactors avoid biofilm removal but generate dilute biomass suspensions that require energy-intensive concentration and dewatering, often through centrifugation (Wada et al., 2023b; Doki et al., 2024). Membrane-assisted systems improve biomass retention and effluent quality but introduce challenges associated with membrane fouling, maintenance, and capital costs (Lu et al., 2019a). As system scale increases, harvesting challenges extend beyond biomass recovery itself. Dewatering, drying, storage, and transportation of biomass can substantially increase operational costs and may become major determinants of overall process feasibility. Consequently, harvesting strategy must be considered alongside reactor design and product objectives rather than as a downstream operation. Despite recent advances, no harvesting approach has yet demonstrated an optimal balance between recovery efficiency, energy demand, operational simplicity, and cost at pilot-scale. Future research should therefore focus on developing scalable, low-energy biomass recovery technologies. Promising approaches include biofilm engineering to facilitate biomass collection, granular biomass formation to improve settling, gravity-assisted sedimentation, coagulation-flocculation, dissolved air flotation, membrane-assisted concentration, and hybrid harvesting systems that combine multiple separation techniques. In addition, integrating biomass recovery with downstream dewatering and product processing will be essential for improving the overall techno-economic feasibility of PPB-based wastewater biorefineries (Cao et al., 2020).

### 8.3 Light distribution and reactor configuration

Light availability emerged as the most consistent engineering constraint across all reviewed pilot-scale PPB systems. As reactor size increases, longer optical paths, higher biomass concentrations, and biofilm accumulation reduce photon penetration and create spatial heterogeneity within the culture. Consequently, reactor performance is governed not only by microbial activity but also by the ability to maintain sufficient light distribution throughout the system (Cañedo and Lizárraga, 2016; Molina Grima et al., 2000). Although the severity of light limitation varies among reactor configurations, a common trend was observed across all pilot-scale studies. Flat-plate photobioreactors generally achieved superior light utilization due to their high surface-to-volume ratios and short optical paths. However, biofilm accumulation progressively reduced light penetration and required periodic biomass removal to maintain productivity (Hülsen et al., 2022a, 2022b; Capson-Tojo et al., 2023b). In contrast, tubular reactors experienced longitudinal light gradients and greater sensitivity to seasonal irradiance, while larger baffled and open systems exhibited spatial heterogeneity caused by uneven light distribution and mixing (Boran et al., 2010, 2012a, 2012b; Adessi et al., 2012; Zhang et al., 2017). Direct comparisons further demonstrate that reactor geometry can influence productivity as strongly as operational conditions. For example, Gebicki et al. (2010) reported higher hydrogen productivity in a flat-panel reactor than in a tubular reactor operated under similar conditions, primarily due to improved light distribution. Similarly, raceway and stirred systems required continuous mixing to maintain an active photic zone and reduce biomass shading (Wada et al., 2023b; Doki et al., 2024). Collectively, the reviewed studies indicate that light limitation intensifies with scale regardless of reactor configuration and remains one of the primary barriers to commercial deployment. Future research should focus on improving light delivery through optimized reactor geometry, advanced mixing strategies, internal illumination systems, optical materials, and dynamic biomass control to maximize photosynthetically active volume while minimizing energy consumption.

### 8.4 Substrate balance, nutrient control, and cost implications

Substrate composition and nutrient balance strongly influence PPB enrichment, biomass productivity, and product formation. While nutrient-rich wastewaters such as poultry-processing and piggery wastewaters can support stable PPB growth with minimal supplementation (Hülsen et al., 2022a, 2022b; Capson-Tojo et al., 2023b), nutrient-deficient streams such as fuel-synthesis wastewater often require pH adjustment, trace elements, vitamins, or additional nutrient sources to sustain growth (Wada et al., 2023b). Similarly, several hydrogen-production studies relied on synthetic substrates, which improve process control but reduce economic feasibility at larger scales (Boran et al., 2010, 2012a, 2012b; Adessi et al., 2012). Nutrient requirements also vary according to the target product. SCP production generally favors sufficient nitrogen availability to maximize biomass growth, whereas hydrogen and PHA production often rely on nutrient-limited conditions that redirect carbon and reducing power toward product formation. Consequently, operating conditions that maximize one product may not be optimal for another. As system scale increases, nutrient supplementation introduces additional costs associated with chemical procurement, transportation, storage, preparation, and dosing. In some cases, the cost of feedstock conditioning and nutrient supplementation may offset a substantial fraction of the value generated through resource recovery. Therefore, maximizing product yield does not necessarily correspond to the most economically viable operating strategy. Feedstock conditioning through fermentation, wastewater blending, nutrient recycling, and the use of nutrient-rich co-substrates can reduce dependence on externally supplied chemicals while improving substrate suitability for PPB cultivation (Almeida et al., 2024; Doki et al., 2024). Future research should prioritize such approaches to improve process robustness, reduce operating costs, and enhance the scalability of PPB-based resource recovery systems.

### 8.5 Knowledge gaps and scale-up needs

Despite encouraging pilot-scale results, several critical knowledge gaps continue to limit the transition of PPB-based wastewater treatment and resource recovery systems toward industrial implementation. Most pilot-scale studies have focused on short- to medium-term operation (Carlozzi and Sacchi, 2001; Boran et al., 2010, 2012a, 2012b; Wada et al., 2023b). Although a few long-term studies have been reported (Lu et al., 2019a; Almeida et al., 2024), the stability of PPB-enriched communities under seasonal fluctuations, variable wastewater composition, shock loading, and prolonged operational stress remains insufficiently understood. In particular, microbial succession, community resilience, and their effects on biomass composition and product yields remain poorly understood under realistic outdoor conditions. Improved understanding of these factors is essential to ensure stable process performance at commercial scale.

Another major limitation is the lack of integrated system-level evaluation. While many pilot studies (Lu et al., 2019a; Hülsen et al., 2022a, 2022b; Capson-Tojo et al., 2023b; Wada et al., 2023b; Almeida et al., 2024) report wastewater treatment efficiencies and product recovery, comprehensive assessments of energy demand, biomass harvesting, nutrient supplementation, downstream processing, and overall economic performance remain scarce. Consequently, it is often difficult to determine whether improvements in product yield justify the additional operational complexity and cost required to achieve them.

Although environmental sustainability will ultimately influence commercial deployment, published life-cycle assessment (LCA) studies specifically evaluating pilot-scale PPB systems remain scarce. Consequently, environmental impacts associated with illumination, biomass harvesting, nutrient supplementation, and downstream processing have not yet been systematically quantified. Future studies integrating reactor performance with LCA and techno-economic assessment (TEA) will be essential for providing a more comprehensive evaluation of PPB technologies.

Digital twin technology offers a complementary approach for overcoming operational challenges that are difficult to investigate experimentally (Tao et al., 2019; Sharma et al., 2022). By integrating mechanistic models with operational data, digital twins can create virtual representations of pilot-scale PPB systems capable of simulating light attenuation, biofilm accumulation, nutrient limitation, biomass growth, temperature fluctuations, and wastewater variability (Nikou et al., 2026). Such models could be used to evaluate different reactor geometries, mixing strategies, harvesting frequencies, and seasonal operating conditions without extensive experimental testing. Digital twins may also support predictive process control by identifying optimal operating conditions, forecasting productivity losses, and providing early warning of process deterioration (Sharma et al., 2022; Nikou et al., 2026). As system size increases and experimental optimization becomes increasingly expensive, these capabilities become particularly valuable.

Although SCP, PHAs, pigments, lipids, and other products have been successfully recovered at pilot-scale, validation of their end-use applications remains extremely limited. Most studies are limited to biomass characterization and product quantification, with little attention given to downstream purification, product qualification, regulatory compliance, or market acceptance. For example, PPB-derived SCP has rarely been evaluated through long-term aquaculture feeding trials, while PHAs recovered from pilot-scale systems have seldom been assessed for polymer properties, processing performance, or commercial applicability. Consequently, technology readiness remains lower than reported treatment and recovery efficiencies may suggest.

Future research should therefore move beyond reactor performance alone and adopt integrated technical, economic, environmental, and product-oriented evaluation frameworks. The combined application of LCA and digital twin technologies, together with long-term operational studies and application-focused product validation, will be essential for reducing scale-up uncertainty and accelerating the commercial deployment of PPB-based wastewater biorefineries.

## 9 Evidence-based decision-support framework for reactor selection

**Figure 2:**
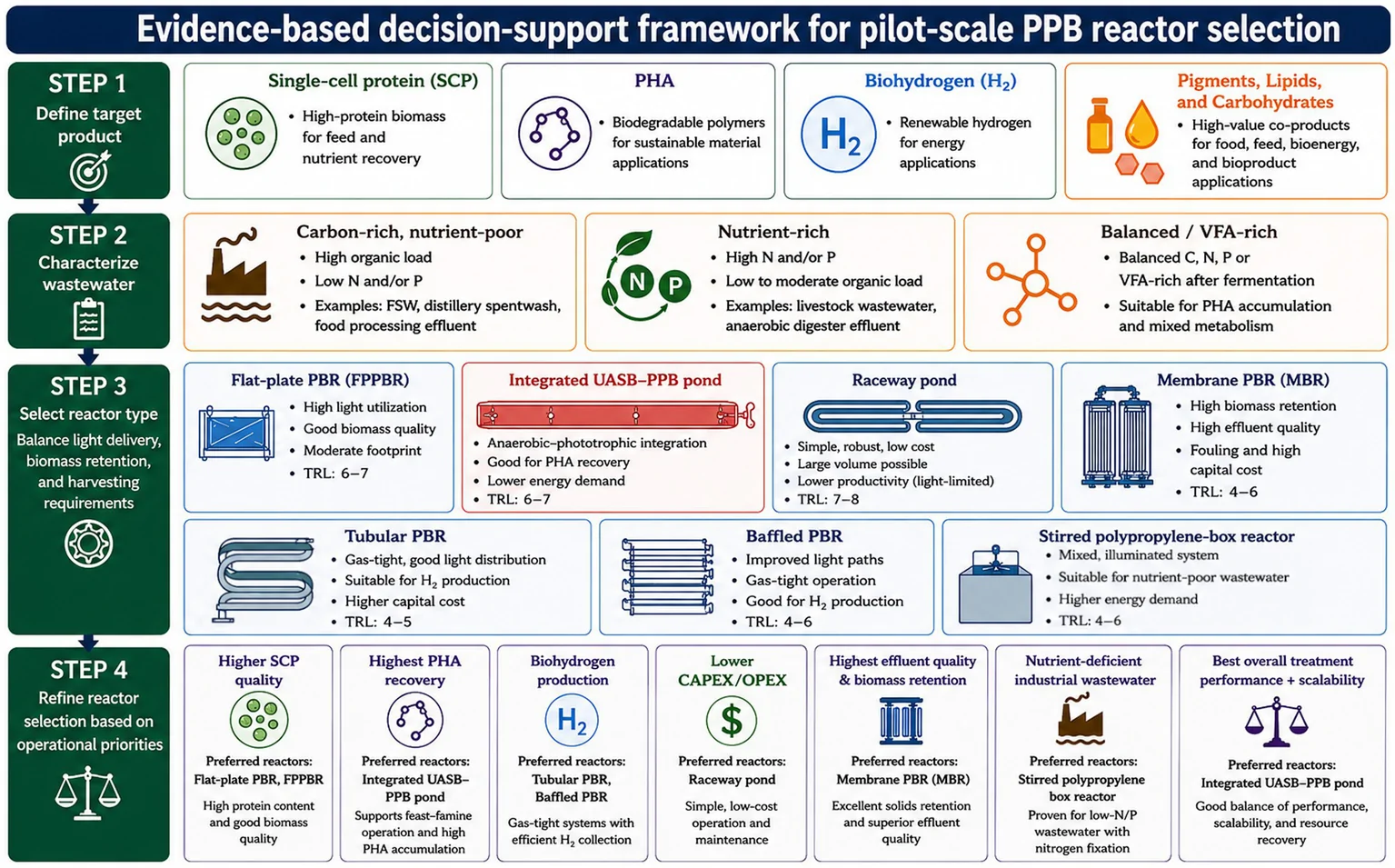
Evidence-based decision-support framework for pilot-scale PPB reactor selection.

The preceding sections demonstrate that pilot-scale performance is governed less by reactor volume than by the alignment between wastewater characteristics, target resource recovery objectives, reactor configuration, and operational priorities. While existing reviews have summarized reactor designs and process performance, none have provided a systematic framework linking wastewater characteristics, target products, reactor selection, and implementation objectives for pilot-scale deployment. To address this gap, the evidence synthesized in this review is translated into a four-step evidence-based decision-support framework (Figure 2): (i) define the target recovery product, (ii) characterize the wastewater and its conditioning requirements, (iii) select the reactor configuration that best balances light delivery, biomass retention, and harvesting requirements, and (iv) refine reactor selection according to operational priorities such as product quality, energy demand, effluent quality, biomass retention, or capital and operating costs. The framework is summarized as a wastewater-reactor-product matrix (Table 3) and complemented by a transparent multi-criteria ranking of reactor configurations (Table 4). Detailed scoring criteria, evidence sources, and calculation procedures are provided in Supplementary Tables S7a–c.

**Table 3:** Wastewater reactor product selection matrix for pilot-scale PPB systems.

Wastewater stream	Key characteristics	Preferred configuration(s)	Primary product	Rationale
Domestic/municipal sewage	Balanced; low-strength; VFA-rich after fermentation	Raceway PBR; UASB–PPB pond	SCP; PHA	Mature open systems; pre-fermentation supplies VFAs
Poultry-processing	Nutrient-rich; high COD/VFA	Flat-plate PBR	SCP	Biofilm enrichment under sunlight; product differentiation
Piggery	Nutrient-rich; high ammonia	Flat-plate PBR (NIR-filtered)	SCP	Selective PPB enrichment; manage ammonia inhibition
Brewery	Carbon-dominated; moderate strength	Membrane PBR	SCP + bioactives	Biomass retention; long-term stability
Fuel-synthesis	Carbon-rich; zero nitrogen; acidic	Stirred polypropylene-box PBR (N-limited)	SCP	Diazotrophic growth; minimal chemical input
Dark-fermenter effluent/defined VFA	VFA- or sugar-rich; defined	Tubular PBR; Baffled PBR	Biohydrogen	Closed, gas-tight; N-limited photofermentation

VFA, volatile fatty acids; COD, chemical oxygen demand; UASB, upflow anaerobic sludge blanket; PPB, purple phototrophic bacteria; SCP, single-cell protein; PHA, polyhydroxyalkanoates; FPPBR, flat-plate photobioreactor; NIR, near-infrared; PBR, photobioreactor; FSW, fuel synthesis wastewater.

Pigments and lipids are recovered as intrinsic co-products of phototrophic biomass across all configurations and therefore inform valorization potential rather than reactor choice.

**Table 4:** Weighted multi-criteria comparative ranking of pilot-scale PPB photobioreactor configurations.

Reactor configuration	Treatment (0.20)	Product (0.20)	Scale-up (0.20)	Energy (0.15)	Op. simplicity (0.15)	Harvesting (0.10)	Weighted /100
Flat-plate PBR	4	5	4	4	3	3	79
Integrated UASB–PPB pond	4	4	5	4	3	2	77
Raceway PBR	4	3	5	4	4	2	76
Membrane PBR	4	4	3	2	2	5	66
Baffled PBR	2	3	3	3	2	3	53
Tubular PBR	2	3	2	3	2	3	49
Stirred polypropylene-box PBR	3	3	2	1	3	2	48

FPPBR, flat-plate photobioreactor; UASB, upflow anaerobic sludge blanket; PPB, purple phototrophic bacteria; PBR, photobioreactor; Op, operational.

The wastewater-reactor-product matrix (Table 3) reveals three practical conclusions. First, SCP is the most versatile and robust resource recovery pathway, having been successfully demonstrated across multiple wastewater types and reactor configurations, including flat-plate, raceway, membrane, and stirred systems. Consequently, reactor selection for SCP production is governed primarily by biomass quality, harvesting requirements, and operational cost rather than wastewater type alone. Second, hydrogen and PHA production require substantially greater process specialization. Hydrogen production is largely restricted to closed, gas-tight systems such as tubular and baffled photobioreactors that enable efficient gas recovery and strict environmental control. In contrast, PHA accumulation depends on carefully controlled carbon availability, nutrient balance, and feast-famine operation, often facilitated through integrated anaerobic-phototrophic configurations. Third, pigments, lipids, and other intracellular metabolites are consistently recovered as co-products of PPB biomass. Their production is therefore influenced more strongly by operating conditions, including irradiance, nutrient availability, and growth mode, than by reactor configuration itself. These products enhance biomass valorization potential but generally do not dictate reactor selection.

To move beyond qualitative comparisons, a semi-quantitative multi-criteria assessment was conducted (Table 4). Seven reactor configurations were evaluated against six weighted criteria: treatment efficiency (0.20), product recovery potential (0.20), scale-up potential (0.20), energy efficiency (0.15), operational simplicity (0.15), and harvesting ease (0.10). Each criterion was scored from 1 (least favorable) to 5 (most favorable), and weighted scores were normalized to a 100-point scale. The scoring provides a general comparison of reactor configurations rather than a universal ranking, as individual applications may prioritize criteria such as effluent quality, biomass retention, or operational simplicity differently depending on treatment objectives. Based on this assessment, flat-plate photobioreactors achieved the highest overall score (79/100), followed by integrated UASB-PPB pond systems (77/100) and raceway ponds (76/100). Membrane photobioreactors ranked moderately (66/100), whereas baffled (53/100), tubular (49/100), and stirred polypropylene-box photobioreactor (48/100) received lower scores due to higher energy demand, increased operational complexity, or specialization toward specific products and substrates.

Importantly, the framework extends beyond reactor ranking by incorporating operational priorities as a final decision layer. While several reactor configurations may be technically suitable for a given wastewater-product combination, the preferred option ultimately depends on the primary implementation objective. For example, flat-plate photobioreactors are most suitable when maximizing SCP quality and biomass value is the priority, whereas integrated UASB-PPB pond systems are preferable for PHA-oriented applications. Raceway ponds are advantageous when minimizing capital and operating costs is the primary objective, while membrane photobioreactors are more appropriate when superior effluent quality and biomass retention are required. Similarly, tubular and baffled photobioreactors remain the preferred options for hydrogen production because their closed configurations facilitate efficient gas recovery and process control. This additional decision layer shifts the framework from a purely technical comparison toward a practical tool for application-driven reactor selection.

The resulting framework is intended to support both researchers and practitioners during early-stage technology selection and process development. By integrating wastewater characteristics, resource recovery objectives, reactor performance, and operational priorities into a single decision pathway, the framework provides a practical basis for selecting pilot-scale PPB technologies. Furthermore, it establishes a foundation for future integration with techno-economic assessment, life-cycle assessment, and digital twin modeling approaches, enabling more comprehensive evaluation of PPB-based wastewater biorefineries as they progress toward commercial implementation. The final reactor selection should also be considered alongside the Technology Readiness Level (TRL) assessment presented in Section 10 (Table 5), as commercial implementation depends not only on technical suitability but also on technology maturity and deployment readiness. However, the proposed evidence-based decision-support framework is derived from the evidence synthesized from the 15 pilot-scale studies included in this review. Additional criteria, such as long-term process stability, robustness to environmental fluctuations, land footprint, maintenance requirements, carbon footprint, and detailed CAPEX and OPEX were not incorporated because these metrics are inconsistently reported across the available pilot-scale studies. Consequently, the wastewater–reactor–product relationships, reactor rankings, and implementation recommendations should be considered evidence-based guidance rather than universally applicable decision rules. Future refinement of the framework should incorporate these criteria as they become more consistently reported in pilot-scale studies. In addition, future studies could strengthen the framework by applying sensitivity analysis and formal multi-criteria decision-making approaches, such as the Analytic Hierarchy Process (AHP), once a larger body of pilot-scale data becomes available. As additional pilot-scale studies encompassing a broader range of wastewaters, reactor configurations, operational conditions, and resource recovery objectives become available, the framework should be further validated and refined.

**Table 5:** Indicative technology-readiness level (TRL) and commercialization status of pilot-scale PPB reactor configurations.

Reactor configuration	TRL	Evidence basis (this review)	Commercialization status	Reference
Raceway PBR	7–8	2,500 L treating real domestic sewage; mature HRAP analog	Closest to deployment for low-cost treatment and SCP	Doki et al. (2024)
Integrated UASB–PPB pond	6–7	2 × 9,600 L outdoor treating real domestic wastewater over two seasons	Demonstration-scale; needs year-round and harvesting validation	Almeida et al. (2024)
Flat-plate PBR	6–7	950–953 L; up to 253 days outdoors treating poultry-processing wastewater	Pilot-validated; biofilm control and harvesting are bottlenecks	Hülsen et al. (2022a), Hülsen et al. (2022b), Capson-Tojo et al. (2023b)
Membrane PBR	5–6	240 L, 440 days continuous operation under artificial illumination	Pilot-scale; fouling and energy limit scale-up	Lu et al. (2019a)
Tubular PBR	5–6	50–90 L pilot-scale reactors for H_2_ production using pure culture and defined substrate	Pilot-scale; pure-culture and synthetic-substrate dependence	Boran et al. (2010), Boran et al. (2012a), Boran et al. (2012b), Adessi et al. (2012)
Baffled PBR	5	4,000 L treating synthetic glucose based medium; strong substrate inhomogeneity	Process-demonstration stage; defined feed	Zhang et al. (2017)
Stirred polypropylene-box PBR	4–5	65 L reactor (60 L working volume) treating fuel-synthesis wastewater under artificial light	Early pilot; primarily a process-optimization tool	Wada et al. (2023b)

TRL, Technology Readiness Level; HRAP, high-rate algal pond; SCP, single-cell protein; UASB, upflow anaerobic sludge blanket; PPB, purple phototrophic bacteria; FPPBR, flat-plate photobioreactor; PBR, photobioreactor; H_2_, hydrogen.

## 10 Technology readiness and commercialization potential

Reactor selection for industrial implementation must consider not only technical performance but also technology maturity and commercial readiness. Pilot-scale demonstration does not necessarily indicate deployment readiness, and the reactor configurations reviewed here occupy different positions on the technology readiness scale. Table 5 presents indicative Technology Readiness Levels (TRLs) assigned according to the largest validated operating volume, duration of operation, use of real wastewater, dependence on synthetic substrates, and reliance on artificial illumination. The detailed scoring methodology and supporting evidence are provided in Supplementary Tables S8a,b.

Three broad maturity tiers can be identified. Open raceway ponds represent the most mature configuration (TRL 7–8), benefiting from established pond-based wastewater treatment infrastructure and relatively low capital and operating costs. However, limitations in light utilization, biomass selectivity, and productivity may restrict their suitability for high-value resource recovery applications (Doki et al., 2024). Integrated UASB-PPB pond systems and flat-plate photobioreactors occupy an intermediate demonstration-ready tier (TRL 6–7). These systems have demonstrated stable outdoor operation at pilot-scale using real wastewater and have shown potential for simultaneous treatment and resource recovery. Nevertheless, challenges related to biomass harvesting, biofilm management, seasonal variability, and downstream processing must be resolved before large-scale deployment (Hülsen et al., 2022a, 2022b; Capson-Tojo et al., 2023b; Almeida et al., 2024). Membrane photobioreactors and tubular photobioreactors remain at the pilot stage of development (TRL 5–6), whereas baffled photobioreactors (TRL 5) and stirred polypropylene-box photobioreactors (TRL 4–5) are at earlier stages of development, largely due to constraints associated with membrane fouling, energy demand, gas management, operational complexity, or dependence on defined media and controlled operating conditions (Boran et al., 2010, 2012a, 2012b; Zhang et al., 2017; Lu et al., 2019a; Wada et al., 2023b).

Despite encouraging technical performance, commercialization barriers extend beyond reactor operation. Most pilot-scale studies focus on treatment efficiency and product recovery, whereas critical aspects such as product standardization, regulatory approval, market acceptance, supply-chain integration, and downstream product processing remain largely unexplored. For example, PPB-derived SCP has not yet been extensively validated through large-scale aquaculture feeding trials, while pilot-scale PHA production has rarely progressed to polymer characterization, product certification, or industrial processing assessment. Consequently, technology readiness for product utilization is generally lower than technology readiness for product generation.

A phased commercialization pathway can therefore be proposed. In the short term, efforts should focus on demonstrating year-round operational stability, reducing harvesting and dewatering costs, improving light-use efficiency, and ensuring consistent product quality under real wastewater conditions. In the medium term, demonstration-scale facilities (approximately 100–1,000 m^3^) should incorporate techno-economic assessment, life-cycle assessment, and digital twin-based process optimization to support data-driven scale-up decisions. Long-term deployment will likely involve integration of PPB systems into circular resource-recovery biorefineries, where wastewater treatment is combined with the production of SCP, PHAs, pigments, hydrogen, and other value-added products. Achieving this transition will require coordinated advances in reactor engineering, product validation, regulatory approval, and market development alongside continued improvements in process economics and environmental performance.

## 11 Economic and operational considerations

The transition of PPB-based wastewater treatment from pilot-scale demonstration to industrial implementation ultimately depends on economic viability and environmental sustainability. Although detailed economic evaluations remain scarce, the reviewed pilot-scale studies allow identification of the principal cost drivers associated with different reactor configurations (Table 6), including illumination, mixing, biomass harvesting, nutrient supplementation, pretreatment requirements, land demand, and capital investment. The evidence supporting this qualitative assessment is provided in Supplementary Table S9.

**Table 6:** Qualitative techno-economic comparison of pilot-scale PPB reactor configurations.

Reactor configuration	Lighting	Mixing/aeration	Harvesting	Land	Relative capital
Flat-plate PBR	Sunlight (≈0 energy)	Low (recirculation)	Biofilm scraping (low–moderate)	Medium	Medium
Raceway PBR	Sunlight + IR supplement	Low (paddlewheel)	Dilute; centrifugation (high)	High	Low
Integrated UASB–PPB pond	Sunlight (≈0 energy)	Low–moderate (famine aeration)	Dilute pond biomass (high)	High	Low–medium
Membrane PBR	Artificial (indoor)	Moderate (membrane pumping)	Membrane retention (low energy)	Low	High (membranes)
Stirred polypropylene-box PBR	Artificial illumination (≈11 W L^-1^)	Moderate (stirring)	Dilute decant (moderate–high)	Low	Low–medium
Tubular PBR	Solar + artificial	Moderate (circulation, gas-tight)	Gas product (biomass retained)	Medium	High (tubing, sealing)
Baffled PBR	Solar fiber + PV-LED	Moderate (inter-chamber flow)	Gas product (biomass retained)	Medium	High (multi-unit plant)

PPB, purple phototrophic bacteria; FPPBR, flat-plate photobioreactor; UASB, upflow anaerobic sludge blanket; PBR, photobioreactor; IR, infrared; PV-LED, photovoltaic-powered light-emitting diode; W L^-1^, watts per litre.

Across all configurations, light delivery and biomass harvesting emerge as the most influential operational cost factors. Systems relying primarily on natural sunlight, such as raceway ponds, flat-plate photobioreactors, and integrated UASB-PPB pond systems, benefit from lower energy consumption but require larger footprints and are more susceptible to seasonal and diurnal fluctuations. In contrast, artificially illuminated systems provide greater process control and productivity but incur substantially higher energy costs. For example, the pilot-scale stirred polypropylene-box photobioreactor reported by Wada et al. (2023b) required approximately 11 W L^-1^ of installed lighting capacity, illustrating the economic challenge associated with artificial illumination at larger scales.

Biomass recovery represents a second major economic bottleneck. Open and suspended-growth systems generally require energy-intensive harvesting and dewatering operations, whereas membrane-assisted systems improve biomass retention but introduce costs associated with membrane fouling, cleaning, and replacement. Additional expenses may arise from biomass drying, storage, transportation, and downstream product processing, particularly when recovered biomass is intended for feed, bioplastic, or biochemical applications.

Nutrient management also has important economic implications. While nutrient-rich wastewaters may support PPB growth with minimal supplementation, nutrient-deficient streams often require pH adjustment, trace elements, vitamins, or additional nutrient sources. At larger scales, the procurement, transportation, storage, preparation, and dosing of these chemicals can significantly increase operating costs. Consequently, strategies that reduce external inputs through wastewater blending, nutrient recycling, or upstream fermentation may reduce operating costs.

Treatment efficiency alone does not necessarily reflect the practical feasibility of pilot-scale PPB systems. Auxiliary operations, including artificial illumination, centrifugation, membrane operation, and chemical supplementation, may substantially increase energy demand and operating costs. Consequently, reactor design, process integration, and energy-efficient operation should be considered alongside treatment performance and resource recovery when evaluating pilot-scale technologies.

## 12 Conclusion

This review provides the first comprehensive synthesis of pilot-scale purple phototrophic bacteria (PPB) technologies for wastewater treatment and resource recovery while translating fragmented evidence into an evidence-based decision-support framework for reactor selection and scale-up. The available evidence demonstrates that successful pilot-scale implementation is governed less by reactor size than by the alignment between wastewater characteristics, target resource recovery objectives, reactor configuration, and operational priorities. Across diverse wastewater streams, PPB consistently enabled simultaneous pollutant removal and recovery of valuable products, including single-cell protein (SCP), polyhydroxyalkanoates (PHAs), hydrogen, pigments, lipids, and other biomass-derived compounds. The proposed framework demonstrates that reactor selection should be application-driven rather than universal. Flat-plate photobioreactors are the preferred configuration for SCP production, integrated UASB–PPB systems are most suitable for PHA recovery, and tubular and baffled photobioreactors remain the most appropriate options for hydrogen production. Overall, flat-plate photobioreactors, integrated UASB–PPB systems, and raceway ponds exhibited the strongest balance between treatment efficiency, resource recovery potential, scalability, and operational feasibility. Despite encouraging pilot-scale progress, commercialization remains constrained by challenges associated with light management, low-energy biomass harvesting, long-term operational stability, nutrient optimization, and downstream product validation. Future research should prioritize demonstration-scale operation, standardized performance reporting, integrated techno-economic and life-cycle assessments, and digital twin-assisted process optimization. Collectively, this review provides a practical foundation for selecting, designing, and scaling PPB-based wastewater biorefineries toward commercial implementation within a circular bioeconomy.

## Glossary

5-ALA: 5-aminolevulinic acid
BChls: Bacteriochlorophylls
BP: Biebl and Pfennig growth medium
CAPEX: Capital expenditure
COD: Chemical oxygen demand
Crts: Carotenoids
C/N/P: Carbon to nitrogen to phosphorus ratio
CWW: Chicken processing wastewater
DAF: Dissolved air flotation
DFE: Dark fermenter effluent
FPPBR: Flat-plate photobioreactor
FSW: Fuel synthesis wastewater
HAU-M1: Mixed photosynthetic bacterial consortium
HRAP: High-rate algal pond
HRT: Hydraulic retention time
IR: Infrared
LDPE: Low-density polyethylene
LCA: Life cycle assessment
LED: Light-emitting diode
MBP: Modified Biebl and Pfennig medium
NIR: Near-infrared
OLR: Organic loading rate
OPEX: Operating expenditure
ORP: Oxidation–reduction potential
PHA: Polyhydroxyalkanoate
PHB: Polyhydroxybutyrate
PPB: Purple phototrophic bacteria
PNSB: Purple non-sulfur bacteria
PSB: Photosynthetic bacteria
PVDF: Polyvinylidene fluoride
RPP: *Rhodopseudomonas palustris* producing growth medium
SCP: Single-cell protein
TCOD: Total chemical oxygen demand
TEA: Techno-economic assessment
TKN: Total Kjeldahl nitrogen
TP: Total phosphorus
TRL: Technology Readiness Level
TSS: Total suspended solids
UASB: Upflow Anaerobic Sludge Blanket
UwTP: Underwater tubular photobioreactor
VFA: Volatile fatty acid
VSS: Volatile suspended solids
