# Supplementary material for: Pilot-scale purple phototrophic bacteria for wastewater treatment and resource recovery: an evidence-based framework for reactor selection and scale-up
Source: Front Microbiol. 2026 Aug 21;17:1917392. doi: 10.3389/fmicb.2026.1917392 (PMC13543492; doi:10.3389/fmicb.2026.1917392)
Supplement: Supplementary file 1 [file Data_Sheet_1.docx]

Pilot-scale purple phototrophic bacteria for wastewater treatment and resource recovery: an evidence-based framework for reactor selection and scale-up

Sultan Shaikh^1*^, Gordon McKay^1^

^1^Division of Sustainable Development, College of Science and Engineering, Hamad Bin Khalifa University, Qatar Foundation, Doha, Qatar

*** Correspondence:**
Sultan Shaikh
sshaikh@hbku.edu.qa

*Supplementary Material*

**Supplementary Tables S1–S6. Supporting tables for the pilot-scale PPB review and evidence base for the decision-support framework**

**TABLE S1 Wastewater Characteristics in Pilot-Scale Studies**

| **Study** | **Wastewater / Medium** | **TCOD (mg/L)** | **TKN (mg/L)** | **NH₄⁺-N (mg/L)** | **TP (mg/L)** | **pH** | **C/N (approx.)** | **C/P (approx.)** |
| --- | --- | --- | --- | --- | --- | --- | --- | --- |
| **Almeida et al. (2024)** | Domestic wastewater | 481 ± 166 | NR | 42 ± 13 | NR | 7.5 ± 0.3 | 11.5 | - |
| **Doki et al. (2024)** | Sewage | 492 ± 70 | NR | 31 ± 5 | NR | 7.14 ± 0.01 | 15.9 | - |
| **Wada et al. (2023)** | Fuel-synthesis wastewater | 2,930 ± 55.86 | 0 ± 0 | 0 ± 0 | 0 ± 0 | 3.15 ± 0.49 | - | - |
| **Capson-Tojo et al. (2023)** | Poultry-processing wastewater | 5,454–5,703 | 206–209 | 26–36 | 33–36 | NR | 26.9 | 161.7 |
| **Hülsen et al. (2022b)** | Poultry-processing wastewater (raw) | 4,288 ± 1,207 | 232 ± 74 | 14 ± 5.4 | 40 ± 13 | NR | 18.5 | 107.2 |
| **Hülsen et al. (2022a)** | Piggery wastewater | 4,130 ± 1,560 | 1,160 ± 284 | 900 ± 240 | 160 ± 68 | 7.95 ± 0.28 | 3.6 | 25.8 |
|  | Chicken-processing wastewater | 3,332 ± 419 | 189 ± 30 | 27 ± 10 | 16.6 ± 14 | NR | 17.6 | 200.7 |
| **Lu et al. (2019)** | Brewery wastewater | 2,200–3,200 | NR | 50.0–70.0 | 5.0–8.0 | 8.0–9.0 | 45.0 | 415.4 |
| **Zhang et al. (2017)** | Glucose-based synthetic medium (10 g/L glucose) | NR | NR | NR | NR | 7 | - | - |
| **Adessi et al. (2012)** | RPP defined medium (malate 4 g/L, glutamate 1 g/L) | NR | NR | NR | NR | 6.93 ± 0.14 | - | - |
| **Gebicki et al. (2010)** | Synthetic medium with acetic acid (1.3 mL L⁻¹) and sodium lactate (0.7 mL L⁻¹ of 50% solution). | NR | NR | NR | NR | 6.3–6.5 | - | - |
| **Boran et al. (2010)** | Modified Biebl and Pfennig medium (acetate 40 mM, Na-glutamate 2 mM, phosphate buffer 22 mM) | NR | NR | NR | NR | 6.4 | - | - |
| **Boran et al. (2012a)** | Modified Biebl and Pfennig medium (acetate 20 mM, Na-glutamate 10 mM, phosphate buffer 22 mM) | NR | NR | NR | NR | 6.4 | - | - |
| **Boran et al. (2012b)** | Thick juice dark- fermenter effluent | 3,700–5,500 | NR | 18 | NR | NR | - | - |
| **Carlozzi et al. (2006)** | Modified van Niel synthetic medium A (2.74 g/L sodium acetate, 0.65 g/L ammonium chloride, and 0.2 g/L yeast extract) (C/N ratio = 4.72)  Modified van Niel synthetic medium B (4 g/L sodium acetate, 0.9 g/L ammonium chloride, and 0.5 g/L yeast extract) (C/N ratio = 5). | NR | NR | NR | NR | 7.0 | - | - |
| **Carlozzi and Sacchi (2001)** | Modified van Niel synthetic medium A | NR | NR | NR | NR | 7.0 ± 0.05 | - | - |

TCOD, total chemical oxygen demand; TKN, total Kjeldahl nitrogen; NH₄⁺-N, ammonium nitrogen; TP, total phosphorus; C/N, carbon-to-nitrogen ratio; C/P, carbon-to-phosphorus ratio; NR, not reported; RPP, *Rhodopseudomonas palustris* producing growth medium.

**N**ote: C/N and C/P ratios were estimated using COD as a proxy for carbon and TKN or NH₄⁺-N for nitrogen, based on data availability. Average values were used for ranges. These ratios are indicative only due to inconsistent reporting across studies. “-” indicates insufficient data for calculation.

| **Study** | **Wastewater Type** | **Pretreatment Steps** | **Purpose/Outcome** | **Quantitative evaluation index** |
| --- | --- | --- | --- | --- |
| **Almeida et al. (2024)** | Domestic wastewater | Desanding, degreasing, sieving (1 mm rotary filter) | Removal of coarse solids and fats | TSS removal |
| **Doki et al. (2024)** | Sewage | Anaerobic fermentation, lamella settling, pH adjustment | VFA enrichment and preparation for PPB growth | VFA concentration, TSS removal, and pH |
| **Wada et al. (2023)** | Fuel-synthesis wastewater | pH adjustment | Preparation for PPB growth | pH |
| **Capson-Tojo et al. (2023)** | Poultry-processing wastewater | DAF, anaerobic fermentation | Solids removal and VFA enrichment | TSS removal, and VFA concentration |
| **Hülsen et al. (2022b)** | Poultry-processing wastewater | DAF, anaerobic fermentation | Solids removal and VFA enrichment | TSS removal, and VFA concentration |
| **Hülsen et al. (2022a)** | Piggery and chicken-processing wastewater | Pre-settling (piggery), acetic acid addition as required | Solids removal and carbon supplementation | TSS removal, and COD concentration |
| **Lu et al. (2019)** | Brewery wastewater | Pre-settling | Removal of suspended solids | TSS removal |
| **Boran et al. (2012b)** | Thick juice dark-fermenter effluent | Autoclaving, dilution, pH adjustment | COD stabilization, acid reduction, improved light penetration | COD reduction, and pH control |

**TABLE S2 Summary of Pretreatment strategies used in Pilot-Scale Studies for Phototrophic Wastewater Treatment**

PPB, purple phototrophic bacteria; VFA, volatile fatty acids; TSS, total suspended solids; COD, chemical oxygen demand; DAF, dissolved air flotation.

**Note:** Quantitative evaluation indices represent key measurable parameters used to assess pretreatment performance. Explicit numerical values are not consistently reported across studies.

TABLE S3 Geometric characteristics of flat-plate (panel) photobioreactors used in pilot-scale PPB studies

| **Parameter** | **Hülsen et al. (2022a)** | **Hülsen et al. (2022b)** | **Capson-Tojo et al. (2023)** | **Gebicki et al. (2010)** |
| --- | --- | --- | --- | --- |
| **Configuration** | Vertical flat-plate | Modular flat-plate | Flat-plate, single channel | Vertical-flat panels |
| **Orientation** | Vertical, east–west | Horizontal, outdoor | Outdoor, vertically illuminated | Vertical |
| **Material** | Acrylic | Clear acrylic | NR | PMMA |
| **Wall thickness** | 0.015 m | NR | NR | 4 mm |
| **Reactor top** | Detachable, non-gas-tight | Open to atmosphere | Foam sheet cover | NR |
| **Total reactor volume** | 60–100 L | 953 L | 953 L | 100 L |
| **Working volume** | 60–100 L | 900 L | 953 L | 100 L |
| **Reactor length** | 1.0 m | 9.8 m | 9.8 m | Panel height 1.0 m |
| **Reactor width / height** | 1.0 m × 1.0 m | Module: 1.22 m × 1.22 m | NR | Panel height 1.0 m |
| **Light path thickness** | 50–100 mm | 80 mm | 80 mm | 25 mm |
| **Number of modules / panels** | Single plate | 8 modules | Continuous | 4 panels |
| **Total Illuminated area** | 1.7 m² per reactor | NR | NR | 2.0 m² per panel |
| **Illuminated Surface-to-volume ratio** | 17–28 m² m⁻³ | 21 m² m⁻³ | 21 m² m⁻³ | NR |
| **Mixing / biofilm control** | NR | NR | Hydro-mechanical mixing & wiping | NR |
| **Light modification** | None | UV–VIS absorbing foil | UV–VIS absorbing foil | None |
| PBR, photobioreactor; FPPBR, flat-plate photobioreactor; PMMA, polymethyl methacrylate; UV, ultraviolet; VIS, visible; NR, not reported | | | | |

**TABLE S4 Geometric characteristics of tubular photobioreactors used in pilot-scale PPB studies**

| **Parameter** | **Adessi et al. (2012)** | **Gebicki et al. (2010)** | **Boran et al. (2012a, 2012b)** | **Boran et al. (2010)** | **(Carlozzi and Sacchi, 2001; Carlozzi et al., 2006)** |
| --- | --- | --- | --- | --- | --- |
| **Reactor configuration** | Parallel | Parallel | Pipe-in-pipe | Parallel tubular | Closed tubular loop |
| **Total / working volume** | 50 L | 65 L | 90 L | 80 L | 53 L |
| **Number of tubes** | 10 | 8 | 9 | 9 | 10 |
| **Tube material** | Pyrex glass | LDPE foil | LDPE | LDPE | Glass |
| **Tube length (each)** | 2.0 m | 1.0 m | 2.35 m | 2.35 m | 2.0 m |
| **Tube internal diameter** | 4.85 cm | 6.0 cm | 6.0 cm | 6.0 cm | 4.8 cm |
| **Tube wall thickness** | NR | 300 µm | 150 µm | NR | NR |
| **Tube orientation** | Horizontal | Inclined (5°) | NR | Inclined (10°) | Horizontal (submerged) |
| **Tube arrangement** | Parallel, PVC U-bends | Parallel, manifold-connected | Two concentric tubes | Parallel tubes | Parallel, PVC U-bends |
| **Total hydraulic length** | 23 m | NR | NR | NR | 20 m |
| **Illuminated surface area** | 1.523 m² | 1.0 m² | NR | 2.0 m² | NR |
| **Ground footprint** | NR | NR | NR | 2.88 m² | 2.0 m² |
| **Cooling system** | Water bath | NR | PVC cooling coils | NR | Submerged water bath |
| PBR, photobioreactor; LDPE, low-density polyethylene; PVC, polyvinyl chloride; NR, not reported. | | | | | |

**TABLE S5 Geometric characteristics of raceway, stirred, membrane, and baffled photobioreactors used in pilot-scale PPB studies**

| **Almeida et al. (2024)** | **Doki et al. (2024)** | **Wada et al. (2023)** | **Lu et al. (2019)** | **Zhang et al. (2017)** |
| --- | --- | --- | --- | --- |
| **Reactor type:** Integrated UASB–PPB pond system  **UASB reactors:** Two parallel units  **UASB working volume:** 20 m³ (each)  **UASB height:** 4.3 m  **UASB diameter:** 2.5 m  **UASB construction material:** Fiberglass  **UASB internal design:** Triphasic (gas–solid–liquid) separator  **Phototrophic reactor type:** Retrofitted high-rate algal pond (HRAP)  **Number of PPB ponds:** Two  **PPB pond working volume:** 9.6 m³ (each)  **PPB pond configuration:** Open raceway-type pond | **Reactor type:** Raceway PBR  **Geometry:** Rectangular basin (5 m × 1 m × 0.5 m)  **Total capacity:** **2.5 m³**  **Working volumes tested:** **500 L, 750 L, and 1000 L**  **Illuminated surface-to-volume ratios:** **10.0, 6.67, and 5.0 m²/m³ (**for 500, 750, and 1000 L, respectively)  **Reactor enclosure (relevant to light control):** Enveloped with **black polythene** (to limit external light interference). | **Reactor type:** Stirred **polypropylene-box** PBR  **Reactor material: Polypropylene**  **Reactor configuration:** **Rectangular tank**  **Total reactor volume:** **65 L**  **Working volume:** **60 L**  **Reactor width:** **56 cm**  **Reactor depth:** **39 cm**  **Reactor height:** **42 cm**  **Reactor headspace:** **Present**  **Reactor sealing:** **Edges tightly strapped to minimize oxygen transfer**  **Mixing system:** **Two vertical standing stirrers** | **Reactor type:** Membrane PBR  **Reactor material:** Transparent plexiglass  **Dimensions:** **160 cm × 30 cm × 50 cm**  **Total volume:** **240 L**  **Working volume:** **200 L**  **Membrane type:** Flat-sheet (PVDF)  **Number of membranes:** **5**  **Membrane arrangement:** Submerged, installed in parallel  **Membrane spacing: 25 cm**  **Module size: 22 cm × 0.6 cm × 32 cm**  **Area per module:** **0.1 m²**  **Membrane pore size:** **0.1 μm** | **Reactor type:** **Baffled PBR**  **Reactor configuration: Multi-chamber baffled reactor**  **Number of chambers: 4**  **Total reactor volume: 4 m³**  **Volume of chamber:** 1 **m³**  **Baffle structure: Fixed internal baffles dividing the reactor into four chambers**  **Chamber function: Each chamber acts as a sequential reaction zone** |

UASB, upflow anaerobic sludge blanket; PPB, purple phototrophic bacteria; HRAP, high-rate algal pond; PVDF, polyvinylidene fluoride.

**TABLE S6 Comparative summary of pilot-scale photobioreactor configurations**

| **Reactor type** | **Light path (mm)** | **S/V ratio (m²/m³)** | **Scale (volume, L)** | **Mixing/flow** | **Key strength** | **Key limitation** |
| --- | --- | --- | --- | --- | --- | --- |
| Flat-plate PBR  (Gebicki et al., 2010; Hülsen et al., 2022a, 2022b; Capson-Tojo et al., 2023) | 25–100 | 17–28 | 60–953 | Limited / wiping | High light utilization | Biofilm handling, scale-up |
| Tubular PBR  (Carlozzi and Sacchi, 2001; Carlozzi et al., 2006; Boran et al., 2010, 2012a, 2012b; Gebicki et al., 2010; Adessi et al., 2012) | 48–60 | NR | 50–90 | Continuous flow / circulation | Uniform light exposure | Operational complexity |
| Raceway PBR  (Doki et al., 2024) | NR | 5–10 | 500–1,000 | Open mixing | Low cost, scalable | Low light efficiency |
| Stirred polypropylene-box PBR  (Wada et al., 2023) | NR | NR | 60 | Mechanical mixing | Good process control | Energy demand |
| Membrane PBR  (Lu et al., 2019) | NR | NR | 200 | Controlled membrane-assisted flow | High biomass retention | Fouling and maintenance |
| Baffled PBR  (Zhang et al., 2017) | NR | NR | 4,000 | Sequential flow | Process stability | Light distribution limitations |
| Integrated UASB–PPB pond  (Almeida et al., 2024) | NR | NR | 9,600 | Open pond flow | Large pilot-scale operation | Limited light control |

PBR, photobioreactor; S/V, surface-to-volume ratio; UASB, upflow anaerobic sludge blanket; PPB, purple phototrophic bacteria; NR, not reported.

**Note:** Light path values and Surface-to-volume ratios are reported where available or described qualitatively.

**Supplementary Methods S7–S9. Methodology supporting the evidence-based decision-support framework (Section 9; Tables 4–6)**

This section documents the methodology supporting the evidence-based decision-support framework presented in the main manuscript (Section 9, Figure 2). It describes the development of the weighted multi-criteria ranking (Table 4), the technology readiness assessment (Table 5), and the qualitative economic comparison (Table 6). All scores, rankings, and indicative values were derived from evidence reported in the reviewed pilot-scale studies and are intended to provide transparent and reproducible comparative guidance rather than absolute performance metrics.

**S7. Methodology for the weighted multi-criteria ranking (Table 4)**

Each of the seven reactor configurations was scored from 1 (least favourable) to 5 (most favourable) against six criteria. Each criterion carries a weight reflecting its importance to industrial translation, and the weights sum to 1.00. The overall score on a 100-point scale is calculated as:

Weighted score (/100) = [ Σ (score*i* × weight*i*) ] ÷ 5 × 100, where score*i* ∈ {1–5} and Σ weight*i* = 1.00.

**TABLE S7a Scoring criteria, weights, and 1–3–5 anchors.**

| **Criterion** | **Weight** | **What it captures** | **Score = 1 (low)** | **Score = 3 (moderate)** | **Score = 5 (high)** |
| --- | --- | --- | --- | --- | --- |
| Treatment efficiency | 0.20 | Demonstrated COD and nutrient removal on real wastewater | Not a treatment focus / defined substrate | 60–75% COD removal | >85% COD plus nutrient (N, P) removal |
| Product recovery | 0.20 | Diversity, quality, and value of recovered products | Single low-value product | One strong product | Multiple high-value products with quality differentiation |
| Scale-up potential | 0.20 | Largest validated volume and maturity of the engineering analogue | <100 L; strong scale barriers | Hundreds of L; moderate maturity | m³-scale demonstrated; mature analogue (e.g. HRAP) |
| Energy efficiency | 0.15 | Reliance on artificial light, mixing, and pumping (higher score = lower energy) | Continuous artificial light + intensive mixing | Artificial light OR pumping | Sunlight-driven; low mixing |
| Operational simplicity | 0.15 | Complexity of control, culture, and maintenance | Pure culture / gas-tight / strict control | Biofilm or fermentation coupling | Simple open operation |
| Harvesting ease | 0.10 | Ease and energy of biomass recovery | Dilute suspension; energy-intensive centrifugation | Biofilm scraping or settling | Membrane retention or self-settling |

COD, chemical oxygen demand; N, nitrogen; P, phosphorus; HRAP, high-rate algal pond.

**TABLE S7b Scoring matrix and weighted result (criterion weights in parentheses).**

| **Reactor configuration** | **Treatment (0.20)** | **Product (0.20)** | **Scale-up (0.20)** | **Energy (0.15)** | **Op. simplicity (0.15)** | **Harvesting (0.10)** | **Weighted /100** |
| --- | --- | --- | --- | --- | --- | --- | --- |
| Flat-plate PBR | 4 | 5 | 4 | 4 | 3 | 3 | **79** |
| Integrated UASB–PPB pond | 4 | 4 | 5 | 4 | 3 | 2 | **77** |
| Raceway PBR | 4 | 3 | 5 | 4 | 4 | 2 | **76** |
| Membrane PBR | 4 | 4 | 3 | 2 | 2 | 5 | **66** |
| Baffled PBR | 2 | 3 | 3 | 3 | 2 | 3 | **53** |
| Tubular PBR | 2 | 3 | 2 | 3 | 2 | 3 | **49** |
| Stirred polypropylene-box PBR | 3 | 3 | 2 | 1 | 3 | 2 | **48** |

PBR, photobioreactor; UASB, upflow anaerobic sludge blanket; PPB, purple phototrophic bacteria; Op., operational.

**Worked example (FPPBR):** (4×0.20) + (5×0.20) + (4×0.20) + (4×0.15) + (3×0.15) + (3×0.10) = 3.95 on the 1–5 scale; 3.95 ÷ 5 × 100 = 79/100. The same arithmetic yields 77, 76, 66, 53, 49, and 48 for the remaining configurations, giving the ranking reported in Table 4.

**TABLE S7c Evidence basis for the scores (key pilot data and source).**

| **Reactor configuration** | **Key pilot-scale evidence supporting the scores** | **Study** |
| --- | --- | --- |
| Flat-plate PBR | 950–953 L; up to 253 d outdoor operation on poultry-processing wastewater; 58% crude protein; biofilm/suspended product differentiation; sunlight-driven; biofilm scraping required. | (Capson-Tojo et al., 2023; Hülsen et al., 2022a, 2022b) |
| Integrated UASB–PPB pond | 9,600 L outdoor ponds coupled to UASB; PHA up to 36% of VSS plus SCP; feast–famine; sunlight-driven; dilute pond biomass; mature HRAP + UASB engineering. | Almeida et al. (2024) |
| Raceway PBR | 2.5 m³, surface-to-volume 10 m² m⁻³; 89% COD, 93% NH₄⁺-N, 81% PO₄ removal (supplemented); SCP 40.3–43.9%; yield 0.88–0.95 g COD g COD⁻¹; open, simple; dilute (0.9 g VSS L⁻¹). | Doki et al. (2024) |
| Membrane PBR | 240 L, 440 d continuous on brewery wastewater; SCP + polysaccharides (17.8%) + coenzyme Q10 (3.9%); membrane retention (easy harvest) but fouling, indoor artificial light, transmembrane-pressure control. | Lu et al. (2019) |
| Baffled PBR | 4 m³ (4×1 m³ chambers), synthetic glucose feed, mixed consortium; H₂ 234–381 mol d⁻¹; solar fibre + PV-LED; strong chamber inhomogeneity; single-product, defined substrate. | Zhang et al. (2017) |
| Tubular PBR | 50–90 L tubular PBRs using pure cultures and defined or fermented substrates; Boran et al. (2010, 2012a, 2012b) reported H_2_ yields of 0.35–0.60 mol H_2_ mol⁻¹ acetic acid, with the yield basis defined as acetic acid fed or consumed depending on the study; Adessi et al. (2012) reported 60.3 L H_2_ over the production period; gas-tight operation and temperature control were important operational considerations. | (Adessi et al., 2012; Boran et al., 2012a, 2012b, 2010) |
| Stirred polypropylene-box PBR | 60 L, fuel-synthesis water (zero N), artificial light ≈680 W (11.3 W L⁻¹); SCP 43%, lipids 25.3%; >70% COD removal (slow); stirred; dilute suspension. | Wada et al. (2023) |

PBR, photobioreactor; UASB, upflow anaerobic sludge blanket; PPB, purple phototrophic bacteria; HRAP, high-rate algal pond; SCP, single-cell protein; PHA, polyhydroxyalkanoates; VSS, volatile suspended solids; COD, chemical oxygen demand; NH₄⁺-N, ammonium nitrogen; PO₄, phosphate; H₂, hydrogen; PV-LED, photovoltaic-powered light-emitting diode.

**S8. Methodology for the technology readiness assessment (Table 5)**

Technology-readiness levels (TRLs) were assigned on the standard 9-point scale using four evidence axes: (i) the largest validated working volume; (ii) operating duration and continuity; (iii) substrate realism (real wastewater versus synthetic or defined media); and (iv) light source and operating environment (outdoor sunlight versus indoor artificial illumination). Pilot-scale phototrophic systems map to TRL 4–8, as defined below.

**TABLE S8a TRL rubric adapted to pilot-scale phototrophic wastewater systems.**

| **TRL** | **Definition (phototrophic wastewater context)** |
| --- | --- |
| TRL 4 | Component/process validated at laboratory scale under controlled conditions. |
| TRL 5 | Process validated in a relevant environment: small pilot (tens of litres) under realistic but still partly controlled conditions, often synthetic substrate or artificial light. |
| TRL 6 | Prototype demonstrated in a relevant environment: pilot of hundreds of litres on real wastewater for an extended period. |
| TRL 7 | System prototype demonstrated in an operational environment: large pilot/demonstration (m³-scale) on real wastewater under outdoor conditions. |
| TRL 8 | System complete and qualified through demonstration; engineering analogue commercially mature. |

TRL, Technology Readiness Level.

**TABLE S8b Evidence basis and assigned TRL per configuration.**

| **Reactor configuration** | **Largest volume** | **Duration** | **Substrate / light** | **TRL** | **Basis (this review)** |
| --- | --- | --- | --- | --- | --- |
| Raceway PBR | 1,000 L | Pilot, multi-volume | Real domestic sewage; Artificial IR supplement | **7–8** | Largest open-system maturity; leverages commercial HRAP engineering; closest to deployment. |
| Integrated UASB–PPB pond | 9,600 L | Two seasons, outdoor | Real domestic WW + molasses; sunlight | **6–7** | Largest validated volume on real WW; needs year-round and harvesting validation. |
| Flat-plate PBR | 953 L | Up to 253 d, outdoor | Real poultry-processing WW; sunlight | **6–7** | Evidence combined across the cited flat-plate pilot studies. |
| Membrane PBR | 240 L | 440 d, continuous | Real brewery WW; indoor artificial light | **5–6** | Long, robust operation but indoor light and fouling limit operational realism. |
| Tubular PBR | 50–90 L | Outdoor, seasonal | Defined/acetate; pure culture; solar+artificial | **5–6** | Outdoor solar H₂ demonstrated but pure-culture, defined-substrate dependence. |
| Baffled PBR | 4 m³ | Continuous, HRT-staged | Synthetic glucose; mixed consortium | **5** | Large volume but synthetic feed and strong inhomogeneity; process-demonstration stage. |
| Stirred polypropylene-box PBR | 60 L | Two runs (≈21 d) | Real fuel-synthesis WW; artificial light | **4–5** | Real industrial WW but small volume and artificial light; primarily an optimization platform. |

TRL, Technology Readiness Level; PBR, photobioreactor; UASB, upflow anaerobic sludge blanket; PPB, purple phototrophic bacteria; HRAP, high-rate algal pond; WW, wastewater; HRT, hydraulic retention time; H₂, hydrogen; IR, infrared.

**S9. Methodology and evidence supporting the qualitative economic comparison (Table 6)**

The qualitative economic comparison presented in Table 6 is supported by the following evidence and illustrative calculations derived from the pilot-scale studies included in this review.

**(1) Volumetric lighting power (artificial-light systems).** For the stirred box reactor, installed lamp power = (4 × 20 W LED strips) + (2 × 100 W) + (2 × 200 W) = 680 W over a 60 L working volume = 11.3 W L⁻¹. Over a 21-day run this is 0.68 kW × 24 h × 21 d ≈ 343 kWh for ≈1 g VSS L⁻¹ × 60 L = 60 g biomass, i.e. on the order of 10³–10⁴ kWh kg⁻¹ biomass, illustrating why continuous artificial illumination is economically challenging at large scale (Wada et al., 2023). Sunlight-driven systems (FPPBR and Integrated UASB–PPB pond) incur essentially zero lighting energy.

**(2) Surface-to-volume ratio and light delivery.** FPPBR provides 21 m² m⁻³ versus 10 m² m⁻³ for the raceway, roughly twice the illuminated area per unit volume, consistent with the higher areal productivity of flat-plate systems and the 0.20 weighting placed on light delivery within scale-up potential (Capson-Tojo et al., 2023; Doki et al., 2024).

**(3) Hydrogen volumetric productivity (baffled reactor).** 381 mol H₂ d⁻¹ in 4 m³ at HRT 24 h = 95.3 mol m⁻³ d⁻¹ ≈ 2.13 m³ H₂ m⁻³ d⁻¹ (STP); 234 mol d⁻¹ at HRT 72 h = 58.5 mol m⁻³ d⁻¹. The HRT–productivity trade-off and pronounced chamber inhomogeneity underlie the moderate scores for this configuration (Zhang et al., 2017).

**(4) Biomass yield.** 0.77 g VSS g COD⁻¹ for the N-limited box reactor (Wada et al., 2023) and 0.88–0.95 g COD*biomass* g COD*removed*⁻¹ for the supplemented raceway (Doki et al., 2024) indicate favourable substrate-to-biomass conversion, supporting the qualitative product-recovery assessment presented in Table 6.

**(5) Harvesting energy.** Centrifugation of dilute suspensions can consume 20–40% of operating energy in comparable systems; membrane retention (Lu et al., 2019) reduces this penalty but adds fouling and capital cost, whereas biofilm systems (FPPBR) permit lower-energy scraping. These relationships provide the evidence supporting the qualitative harvesting assessment presented in Table 6.

**TABLE S9 Evidence basis for the qualitative economic comparison of reactor configurations (qualitative ratings in Table 6 follow from these data).**

| **Reactor configuration** | **Lighting basis** | **Mixing / aeration** | **Harvesting basis** | **Land** | **Relative capital** |
| --- | --- | --- | --- | --- | --- |
| Flat-plate PBR | Sunlight (≈0 energy) | Low (recirculation) | Biofilm scraping (low–moderate) | Medium | Medium |
| Raceway PBR | Artificial | Low (paddlewheel) | Dilute, centrifugation (high) | High | Low |
| Integrated UASB–PPB pond | Sunlight (≈0 energy) | Low–moderate (famine aeration) | Dilute pond biomass (high) | High | Low–medium |
| Membrane PBR | Artificial (indoor) | Moderate (membrane pumping) | Membrane retention (low energy) | Low | High (membranes) |
| Stirred polypropylene-box PBR | Artificial ≈680 W (11.3 W L⁻¹) | Moderate (stirring) | Dilute decant (moderate–high) | Low | Low–medium |
| Tubular PBR | Solar + artificial | Moderate (circulation, gas-tight) | Gas product (biomass retained) | Medium | High (tubing, sealing) |
| Baffled PBR | Solar fibre + PV-LED | Moderate (inter-chamber flow) | Gas product (biomass retained) | Medium | High (multi-unit plant) |

PBR, photobioreactor; UASB, upflow anaerobic sludge blanket; PPB, purple phototrophic bacteria; IR, infrared; PV-LED, photovoltaic-powered light-emitting diode; W L⁻¹, watts per litre.

**References**

Adessi, A., Torzillo, G., Baccetti, E., and De Philippis, R. (2012). Sustained outdoor H 2 production with Rhodopseudomonas palustris cultures in a 50 L tubular photobioreactor. *Int. J. Hydrogen Energy* 37, 8840–8849. doi: 10.1016/j.ijhydene.2012.01.081

Almeida, J. R., León, E. S., Rogalla, F., Fradinho, J. C., Oehmen, A., and Reis, M. A. M. (2024). Polyhydroxyalkanoates production in purple phototrophic bacteria ponds: A breakthrough in outdoor pilot-scale operation. *Science of The Total Environment* 912, 168899. doi: 10.1016/j.scitotenv.2023.168899

Boran, E., Özgür, E., Van Der Burg, J., Yücel, M., Gündüz, U., and Eroglu, I. (2010). Biological hydrogen production by Rhodobacter capsulatus in solar tubular photo bioreactor. *J. Clean. Prod.* 18, S29–S35. doi: 10.1016/j.jclepro.2010.03.018

Boran, E., Özgür, E., Yücel, M., Gündüz, U., and Eroglu, I. (2012a). Biohydrogen production by Rhodobacter capsulatus Hup− mutant in pilot solar tubular photobioreactor. *Int. J. Hydrogen Energy* 37, 16437–16445. doi: 10.1016/J.IJHYDENE.2012.02.171

Boran, E., Özgür, E., Yücel, M., Gündüz, U., and Eroglu, I. (2012b). Biohydrogen production by Rhodobacter capsulatus in solar tubular photobioreactor on thick juice dark fermenter effluent. *J. Clean. Prod.* 31, 150–157. doi: 10.1016/J.JCLEPRO.2012.03.020

Capson-Tojo, G., Zuo Meng Gan, A., Ledezma, P., Batstone, D. J., and Hülsen, T. (2023). Resource recovery using enriched purple phototrophic bacteria in an outdoor flat plate photobioreactor: Suspended vs. attached growth. *Bioresour. Technol.* 373, 128709. doi: 10.1016/j.biortech.2023.128709

Carlozzi, P., Pushparaj, B., Degl’Innocenti, A., and Capperucci, A. (2006). Growth characteristics of Rhodopseudomonas palustris cultured outdoors, in an underwater tubular photobioreactor, and investigation on photosynthetic efficiency. *Appl. Microbiol. Biotechnol.* 73, 789–795. doi: 10.1007/s00253-006-0550-z

Carlozzi, P., and Sacchi, A. (2001). Biomass production and studies on Rhodopseudomonas palustris grown in an outdoor, temperature controlled, underwater tubular photobioreactor. *J. Biotechnol.* 88, 239–249. doi: 10.1016/S0168-1656(01)00280-2

Doki, M. M., Mehta, A. K., Chakraborty, D., Ghangrekar, M. M., Dubey, B. K., Alloul, A., et al. (2024). Recovery of purple non-sulfur bacteria-mediated single-cell protein from domestic wastewater in two-stage treatment using high rate digester and raceway pond. *Bioresour. Technol.* 413, 131467. doi: 10.1016/j.biortech.2024.131467

Gebicki, J., Modigell, M., Schumacher, M., Van Der Burg, J., and Roebroeck, E. (2010). Comparison of two reactor concepts for anoxygenic H2 production by Rhodobacter capsulatus. *J. Clean. Prod.* 18, S36–S42. doi: 10.1016/J.JCLEPRO.2010.05.023

Hülsen, T., Stegman, S., Batstone, D. J., and Capson-Tojo, G. (2022a). Naturally illuminated photobioreactors for resource recovery from piggery and chicken-processing wastewaters utilising purple phototrophic bacteria. *Water Res.* 214, 118194. doi: 10.1016/j.watres.2022.118194

Hülsen, T., Züger, C., Gan, Z. M., Batstone, D. J., Solley, D., Ochre, P., et al. (2022b). Outdoor demonstration-scale flat plate photobioreactor for resource recovery with purple phototrophic bacteria. *Water Res.* 216, 118327. doi: 10.1016/j.watres.2022.118327

Lu, H., Peng, M., Zhang, G., Li, B., and Li, Y. (2019). Brewery wastewater treatment and resource recovery through long term continuous-mode operation in pilot photosynthetic bacteria-membrane bioreactor. *Science of The Total Environment* 646, 196–205. doi: 10.1016/J.SCITOTENV.2018.07.268

Wada, O. Z., Vincent, A., McKay, G., and Mackey, H. R. (2023). Converting Fuel-Synthesis Process Water to Aquaculture Feed by Purple Non-Sulfur Bacteria. *Chem. Eng. Technol.* 46, 1126–1133. doi: 10.1002/ceat.202200535

Zhang, Q., Lu, C., Lee, D. J., Lee, Y. J., Zhang, Z., Zhou, X., et al. (2017). Photo-fermentative hydrogen production in a 4 m3 baffled reactor: Effects of hydraulic retention time. *Bioresour. Technol.* 239, 533–537. doi: 10.1016/J.BIORTECH.2017.05.067
